# Efficacy and safety of T-DXd versus Other HER2-targeted therapies in second-line and later settings for HER2-positive metastatic breast cancer: A Bayesian network meta-analysis

**DOI:** 10.1371/journal.pone.0358540

**Published:** 2026-09-29

**Authors:** Yu Xiao, Jing Fu, Juan Li, Gang Hu

**Affiliations:** 1 Sichuan Academy of Social Sciences, Chengdu, Sichuan, China; 2 Department of Breast Surgery, Sichuan Provincial People’s Hospital, University of Electronic Science and Technology of China, Chengdu, Sichuan, China; Mayo Clinic Cancer Center, UNITED STATES OF AMERICA

## Abstract

**Background:**

HER2-positive advanced breast cancer remains challenging after trastuzumab-based therapy because available regimens differ in survival, response, and toxicity. We compared therapies used in second-line and later settings.

**Methods:**

This PRISMA-NMA-compliant review was registered with PROSPERO (CRD420251244047). PubMed, Embase, Web of Science, and CENTRAL were searched through July 24, 2026. Phase II or III randomized controlled trials after trastuzumab-based therapy were eligible. Bayesian network meta-analysis summarized PFS and OS as hazard ratios and confirmed ORR and safety outcomes as odds ratios. Random-effects models were used where heterogeneity was estimable; fixed-effect Bayesian models were used for the sparse SAE and ILD/pneumonitis networks. DoR was analyzed exploratorily using a fixed-effect Bayesian normal model after prespecified conversion of arm-level summary statistics; TTP was not quantitatively synthesized when the required summary measures were unavailable or clinically non-equivalent.

**Results:**

Sixteen reports representing seven independent randomized trial families and 3,339 unique randomized participants were included. Relative to T-DXd, T-DM1 had less favorable PFS (HR 3.30, 95% CrI 2.06–5.28) and OS (HR 1.52, 1.19–2.01). However, T-DXd was not represented in the second-line-only OS network or the Asia-dominant PFS/OS networks; therefore, its survival ranking reflects evidence primarily from mixed-line, non-Asia-dominant trials. T-DXd produced higher confirmed ORR than Lap-Cap (OR 7.43, 3.80–13.16), PTN-Cap (OR 3.35, 1.24–8.19), and T-DM1 (OR 6.31, 3.98–10.32). DoR showed no clear between-treatment difference in the EMILIA/NALA network, and TTP could not be quantitatively synthesized. Any-grade AE estimates did not indicate a safety advantage for T-DXd. SAE comparisons were also inconclusive: T-DM1 vs Lap-Cap OR 0.90 (0.67–1.21) and T-DXd vs T-DM1 OR 1.30 (0.87–1.93). T-DXd had higher ILD/pneumonitis odds than T-DM1 (OR 5.55, 2.79–12.16); LVD differences were unclear.

**Conclusion:**

Within the connected overall networks, T-DXd showed a favorable survival profile and the highest confirmed ORR, although its survival ranking was not estimable in the second-line-only OS or Asia-dominant survival networks. Its increased ILD/pneumonitis risk relative to T-DM1 is clinically important. Indirect survival comparisons and sparse safety networks require cautious interpretation.

## Introduction

HER2-positive unresectable, locally advanced, or metastatic breast cancer (MBC), particularly after progression on trastuzumab-based therapy or early relapse following adjuvant trastuzumab treatment, represents a significant clinical challenge. This subtype of breast cancer is characterized by the overexpression of the human epidermal growth factor receptor 2 (HER2) protein, which promotes the growth of cancer cells. Historically, HER2-positive breast cancer has been associated with a poor prognosis; however, the advent of anti-HER2 agents has substantially improved outcomes, transforming it into a treatable, albeit generally incurable, chronic condition [1,2]. In this context, optimizing treatment sequencing to balance survival, disease control, and quality of life is essential. A rigorous comparative evaluation of modern HER2-targeted regimens is therefore critical to inform clinical decision-making, guideline development, and health policy.

Despite the availability of multiple effective second-line and later therapies, no single regimen has been established as universally optimal. Available options differ substantially in mechanisms of action, administration routes, efficacy, and toxicity profiles, complicating treatment selection [3]. Traditional pairwise meta-analyses are inadequate for comparing all available regimens simultaneously or for generating a hierarchical ranking of treatments that have not been directly compared. This presents an opportunity for network meta-analysis (NMA) to integrate both direct and indirect evidence, thereby offering a comprehensive comparison and ranking of available treatment options [4].

Several pivotal randomized controlled trials (RCTs) have demonstrated the efficacy of individual regimens. The EMILIA trial showed that trastuzumab emtansine (T-DM1) improved overall survival (OS) compared with capecitabine plus lapatinib in previously treated HER2-positive advanced breast cancer (ABC) [5]. Subsequent studies demonstrated the superiority of trastuzumab deruxtecan (T-DXd) over T-DM1 in the second-line setting, with sustained OS benefit [6]. The HER2CLIMB trial demonstrated that the addition of tucatinib to trastuzumab and capecitabine significantly improved progression-free survival (PFS) and OS compared to placebo [7]. In addition, pyrotinib plus capecitabine and neratinib plus capecitabine have each demonstrated superior efficacy over lapatinib-based regimens in randomized trials [8].

However, the absence of direct head-to-head comparisons among many of these key regimens makes it difficult to determine their relative positioning within treatment sequences. Previous meta-analyses have largely relied on pairwise comparisons and have not provided integrated treatment hierarchies or jointly evaluated efficacy and detailed safety outcomes within a single framework [9]. This leaves a critical evidence gap for real-world sequencing decisions, highlighting the need for more comprehensive comparative analyses [10]. Although the direct T-DXd versus T-DM1 benefit has already been established, the PFS and OS positioning of T-DXd relative to several other regimens remains dependent on indirect evidence.

To address these gaps, this study conducts a systematic review and NMA of phase II and III RCTs evaluating T-DXd, T-DM1, and key tyrosine kinase inhibitor-based capecitabine regimens (tucatinib, lapatinib, neratinib, with or without pyrotinib) in adults with HER2-positive unresectable locally advanced or metastatic breast cancer who progressed after trastuzumab-based therapy or relapsed within 12 months of adjuvant trastuzumab. By synthesizing direct and indirect evidence, this analysis compares regimens across standardized efficacy and safety endpoints, estimates relative effects on PFS and OS where available, and ranks treatments based on efficacy, durability of response, and toxicity profiles. Safety analyses focus on any-grade adverse events (AEs), serious adverse events (SAEs), and clinically relevant organ-specific toxicities. For PFS and OS, quantitative synthesis was limited to interventions belonging to a connected outcome-specific network. The eligible tucatinib comparison formed a disconnected component and did not contribute to the connected PFS or OS estimates. The overarching aim is to generate an integrated benefit-risk hierarchy to support clinical decision-making and treatment sequencing in the post-trastuzumab setting, while identifying residual uncertainties to guide future trials and real-world research.

## Materials and methods

### Registry information

This systematic review and NMA was registered with PROSPERO under registration number CRD420251244047. Ethical approval and informed consent were not required for this study. The results of the NMA were reported in strict compliance with the Preferred Reporting Items for Systematic Reviews and Meta-Analyses for Network Meta-Analyses (PRISMA-NMA) guidelines [11,12].

### Sources

A systematic literature search was conducted independently by two reviewers (Y.X. and J.F.) in PubMed, Embase (Embase.com), Web of Science Core Collection, and the Cochrane Central Register of Controlled Trials (CENTRAL) from database inception through July 24, 2026. The searches retrieved 228 records from PubMed, 217 from Embase, 1,627 from Web of Science, and 1,519 from CENTRAL. No language restrictions were applied, and all 16 included reports were published in English. Conference abstracts and proceedings were retrieved by the broad searches but were not eligible for inclusion as full-text reports. Complete search strategies for all databases are provided in S1 Appendix. Disagreements were resolved through discussion or arbitration by a third reviewer (G.H.).

### Inclusion and exclusion criteria

Studies were included if they met the following criteria: (1) Population: adults (≥18 years) with histologically confirmed HER2-positive (immunohistochemistry 3+ or fluorescence in situ hybridization-positive), unresectable locally advanced or metastatic breast cancer; (2) Prior therapy: progression after trastuzumab-based therapy for advanced disease, including relapse within 12 months after adjuvant trastuzumab-based therapy; (3) Interventions and comparators: randomized comparisons connecting at least two prespecified treatment nodes, including T-DXd, T-DM1, tucatinib-trastuzumab-capecitabine, pyrotinib-capecitabine (PTN-Cap), lapatinib-capecitabine (Lap-Cap), and neratinib-capecitabine (Ner-Cap); (4) Outcomes: at least one prespecified efficacy or safety outcome; and (5) Study design and report type: phase II or III randomized controlled trials published as full-text articles. Exclusion criteria were nonrandomized or single-arm studies; populations outside the advanced or metastatic post-trastuzumab setting; interventions or comparisons that did not connect the prespecified outcome-specific network; conference abstracts, proceedings, protocols, reviews, editorials, letters, or notes; duplicate or companion reports without a new extractable endpoint; and reports with unavailable essential outcome data. Multiple reports from the same trial were linked and were not double-counted. For PFS and OS, trials were classified as second-line-only or mixed second- and later-line populations.

### Study selection

Two reviewers independently screened titles and abstracts (Y.X. and J.L.) and assessed potentially eligible full texts (J.F. and Y.X.). Duplicate records were removed in EndNote X9. Reports were linked at the trial level; when multiple reports described the same trial, the most complete or most recent outcome-specific report was used, and companion reports without a new extractable endpoint were excluded. Publication-level reports were mapped to their parent trial families. For each outcome, only one report from each independent randomized trial family was used, and participant totals were counted once per trial family. Disagreements were resolved by consensus or by a third reviewer (G.H.). Extracted study characteristics included author, publication year, sample size, study duration, geographic region, treatment setting, and baseline clinical characteristics.

### Data analysis

#### Data extraction and processing.

Data were extracted independently by two reviewers (J.F. and J.L.) using a standardized form. Extracted variables included study location; HER2 assessment; disease status; prior therapies; line of therapy; prior pertuzumab exposure; baseline brain metastases; hormone receptor status; ECOG performance status; visceral and liver involvement; treatment regimens and dosing; follow-up; and efficacy and safety outcomes. For binary outcomes, event definitions followed the original trial reports: confirmed response was coded as the event for ORR, whereas occurrence of toxicity was coded as the event for adverse-event outcomes. DoR and TTP were extracted on a continuous time scale. For the exploratory DoR analysis, only reports with a median and both limits of the 95% CI available for each treatment arm were converted using the prespecified approximation mean ≈ median and SE ≈ (upper CI – lower CI)/(2 × 1.96), with arm-level SD = SE × sqrt(n). Reports with a non-estimable or not-reached CI limit were not converted. Reported HRs and 95% confidence intervals (CIs) were extracted for PFS and OS and transformed as log(HR) = ln(HR), with SE[log(HR)] = [ln(upper 95% CI) – ln(lower 95% CI)]/(2 × 1.96). Disagreements in extraction were resolved by consensus or adjudication by G.H.

### Outcomes

Primary outcomes were PFS and OS. PFS was defined, according to the original trial, as the time from randomization or treatment initiation to radiographic progression or death from any cause, whichever occurred first. OS was the time from randomization or treatment initiation to death from any cause. Secondary outcomes were confirmed ORR, DoR, TTP, any-grade adverse events (AEs), serious adverse events (SAEs), interstitial lung disease (ILD) or pneumonitis, and left ventricular dysfunction (LVD). Outcome definitions reported by the original trials were retained, and clinically different safety endpoints were not combined.

### Study risk-of-bias assessment

Risk of bias was assessed independently by two reviewers (Y.X. and J.L.) using the original Cochrane risk-of-bias tool for randomized trials (RoB 1). Seven domains were evaluated: random sequence generation, allocation concealment, blinding of participants and personnel, blinding of outcome assessment, incomplete outcome data, selective reporting, and other bias. Each domain was rated as low, unclear, or high risk. Disagreements were resolved by consensus or by a third reviewer (G.H.) [13].

### Synthesis methods

#### Data analysis.

Analyses were conducted in R version 4.6.1. HRs were used for PFS and OS; ORs for confirmed ORR and binary safety outcomes; and MDs for DoR. For ORR, OR > 1 indicated a higher response probability; for safety outcomes, OR < 1 indicated fewer adverse events. Bayesian estimates were reported with 95% CrIs. Random-effects Bayesian models were used where between-study heterogeneity could be estimated with reasonable support. The SAE network had only one residual degree of freedom for heterogeneity, and the ILD/pneumonitis network contained only two trials with a zero-event arm; these two safety outcomes therefore used fixed-effect Bayesian binomial-logit models with weakly informative Normal(0, 2.5^2) priors on treatment log-ORs and study baselines. The DoR evidence comprised two trial families in a three-node tree and was analyzed with an exploratory fixed-effect Bayesian normal model after the prespecified median/CI conversion. Frequentist Q, tau², and I² diagnostics were generated only where residual degrees of freedom existed and were not used to characterize heterogeneity in very sparse networks.

### Geometry of the network

Outcome-specific network plots were generated to show direct comparisons. Bayesian models were fit by Markov chain Monte Carlo simulation using gemtc for the primary and standard secondary models and JAGS/coda for the sparse fixed-effect SAE, ILD/pneumonitis, and DoR models. Convergence was assessed using trace plots and Brooks-Gelman-Rubin potential scale reduction factors [14]. For continuous secondary outcomes reported as medians, conversion was permitted only when both arms had finite lower and upper 95% confidence limits; the reported median was used as an approximate mean, SE as (upper – lower)/(2 × 1.96), and SD as SE × √n. If required bounds were unavailable or the endpoint was clinically distinct from the target outcome, quantitative synthesis was not performed.

### Inconsistency assessment

Global inconsistency was assessed only for networks in which an alternative inconsistency model was estimable. Local inconsistency was assessed by node splitting only when a closed loop allowed the same comparison to be estimated from direct and indirect evidence. When no closed loop was present, node splitting was not estimable and no local inconsistency P value was reported. Clinical transitivity was assessed by comparing line of therapy, geographic region, prior pertuzumab exposure, baseline brain-metastasis prevalence, eligibility criteria, follow-up, and treatment era across direct comparisons [15,16].

### Rank probabilities of the interventions

Rank probabilities and SUCRA were summarized for Bayesian networks where ranking was estimable. Higher values indicated greater efficacy for efficacy outcomes and a lower probability of an event for safety outcomes. Rankings were not interpreted in isolation and were de-emphasized for sparse safety networks; conclusions were based primarily on relative effects, interval precision, network connectivity, risk of bias, and clinical transitivity [12,17].

### Additional analyses

Exploratory study-level subgroup analyses for PFS and OS were prespecified according to line of therapy, geographic region, prior pertuzumab exposure, and baseline brain-metastasis prevalence. These were trial-level, not patient-level, subgroup analyses. Sensitivity analyses re-fitted estimable networks after exclusion of reports judged at high overall risk of bias, under alternative continuity-correction assumptions for binary outcomes, and in leave-one-study analyses when network connectivity was retained. Because every quantitative outcome network contained fewer than 10 independent studies, comparison-adjusted funnel-plot and Egger testing was not used for inferential interpretation [18].

### Certainty-of-evidence assessment

Certainty of evidence was assessed at the comparison level using the GRADE domains for direct, indirect, and network estimates: risk of bias, inconsistency, indirectness, imprecision, and publication bias. Indirect evidence was downgraded when no direct randomized comparison was available. Imprecision was downgraded by one or two levels according to whether the 95% interval crossed the null and the breadth of clinically important benefit and harm; sparse or zero-event estimates received an additional downgrade under other considerations when they produced unstable or extremely wide intervals. Publication-bias certainty was not downgraded solely on the basis of underpowered small-study-effect tests. Network certainty was not rated higher than the most credible contributing evidence, and treatment ranking did not increase certainty [19]. Detailed judgments are reported in S2 Appendix (S2-S10 Tables).

## Results

### Study selection

The database searches identified 3,591 records: PubMed (n = 228), Embase (n = 217), Web of Science (n = 1,627), and CENTRAL (n = 1,519). After removal of 742 duplicates, 2,849 records were screened. Of these, 2,817 were excluded: ineligible publication type or not an article (n = 1,214; conference abstracts or proceedings, n = 612; reviews, systematic reviews, or meta-analyses, n = 585; editorials, letters, comments, or notes, n = 16; trial protocol, n = 1), wrong population or disease (n = 97), not an advanced or metastatic treatment setting (n = 466), target regimen absent (n = 153), no evidence of randomization in the title or abstract (n = 288), or the target drug was mentioned without an eligible direct network comparison (n = 599). Thirty-two full-text articles were assessed. Sixteen were excluded because prior-treatment or advanced-disease criteria were not met (n = 4), the comparison did not connect the prespecified network (n = 4), random allocation was not confirmed (n = 3), the report was a companion publication without a new extractable endpoint (n = 3), or prespecified efficacy or safety outcomes were unavailable (n = 2). Sixteen eligible reports representing seven independent randomized trial families were included in the qualitative synthesis and outcome-specific NMA (Fig 1).

**Fig 1:**
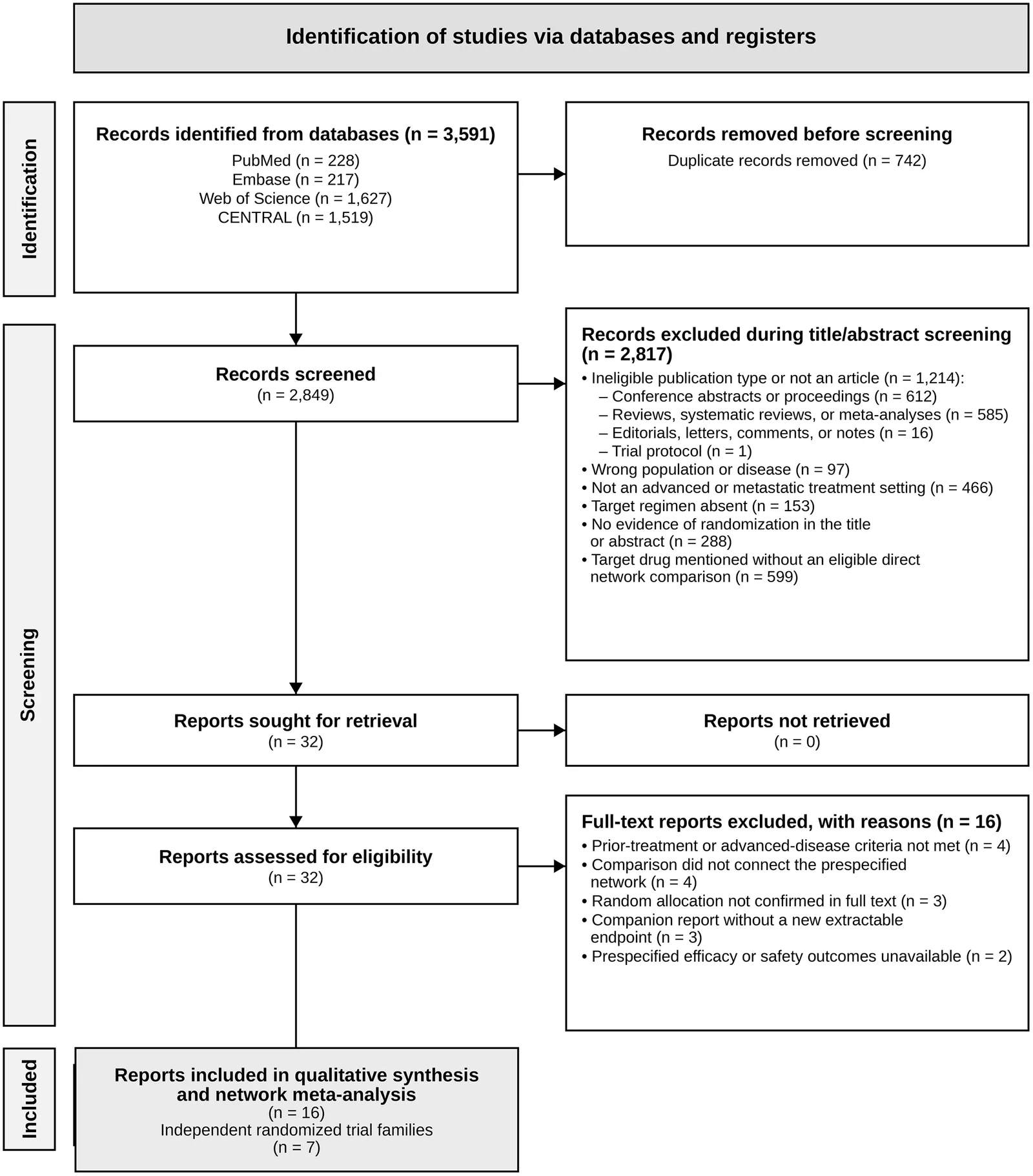
PRISMA 2020 flow diagram of study selection. Flow diagram showing database identification, removal of 742 duplicates, screening of 2,849 records, full-text assessment of 32 reports, and final inclusion of 16 reports representing seven independent randomized trial families, with detailed reasons and counts for exclusion at each stage.

### Study characteristics

The 16 reports represented seven independent randomized trial families and 3,339 unique randomized participants. Study/report identifiers, trial-family mapping, and risk-of-bias judgments are summarized in S1 Table in S2 Appendix. Line of therapy, geographic region, prior pertuzumab exposure, baseline brain-metastasis prevalence, and treatment era varied across trials and were evaluated as potential threats to clinical transitivity when interpreting indirect PFS and OS estimates.

### Risk of bias

Using the Cochrane RoB 1 tool, most reports were judged at low risk for random sequence generation and allocation concealment. Blinding of participants and personnel was frequently unclear, reflecting the open-label design of several oncology trials. One report (Moy-2021) was rated at high risk for blinding of outcome assessment, and several reports had unclear risk for incomplete outcome data or other bias (Figs 2 and 3). Sensitivity analyses excluding reports at high overall risk of bias were conducted where network connectivity permitted.

**Fig 2:**
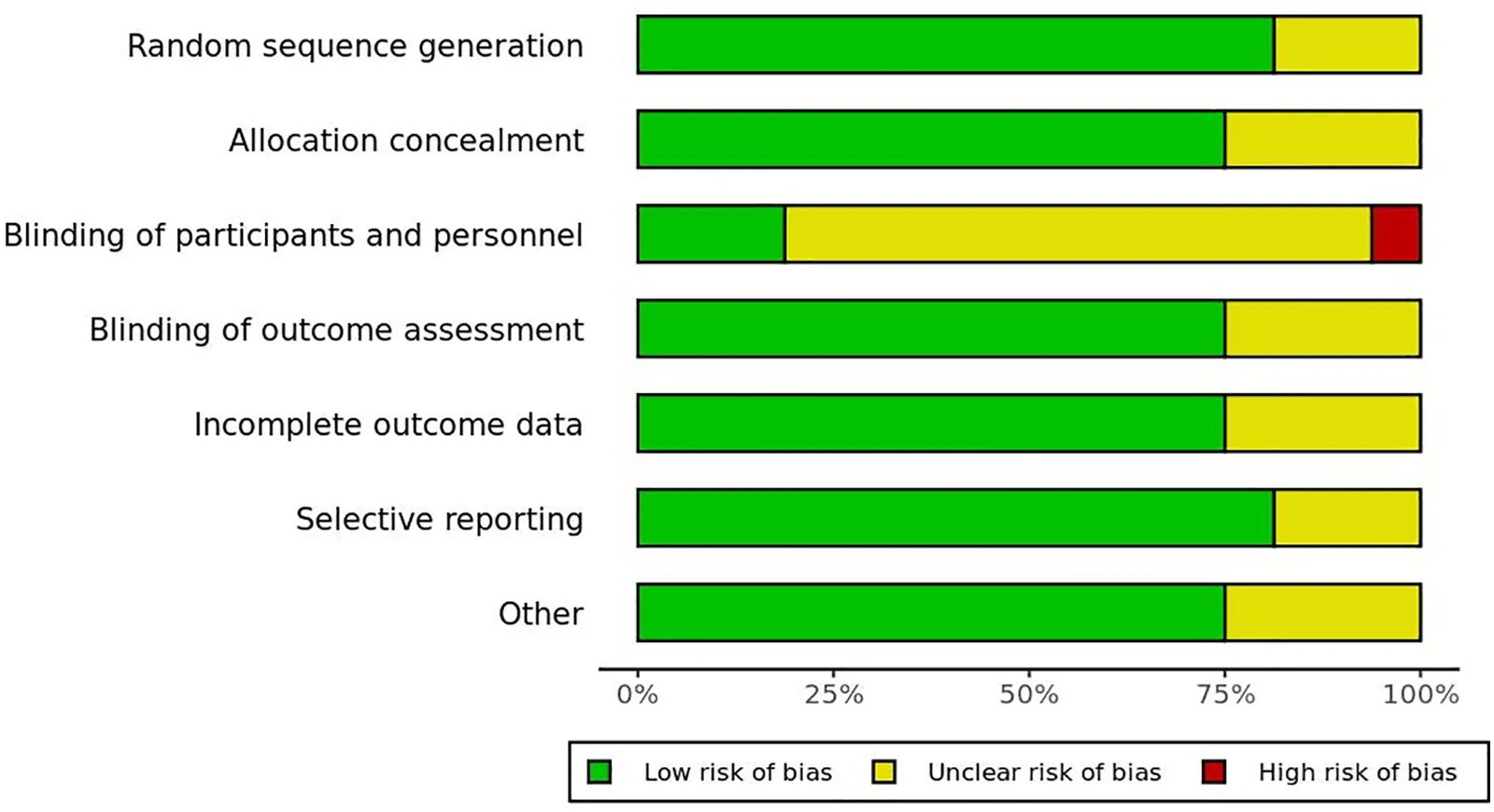
Overall risk of bias across included trial reports. Proportions of included reports rated as low risk (green), unclear risk (yellow), or high risk (red) in each Cochrane RoB 1 domain.

**Fig 3:**
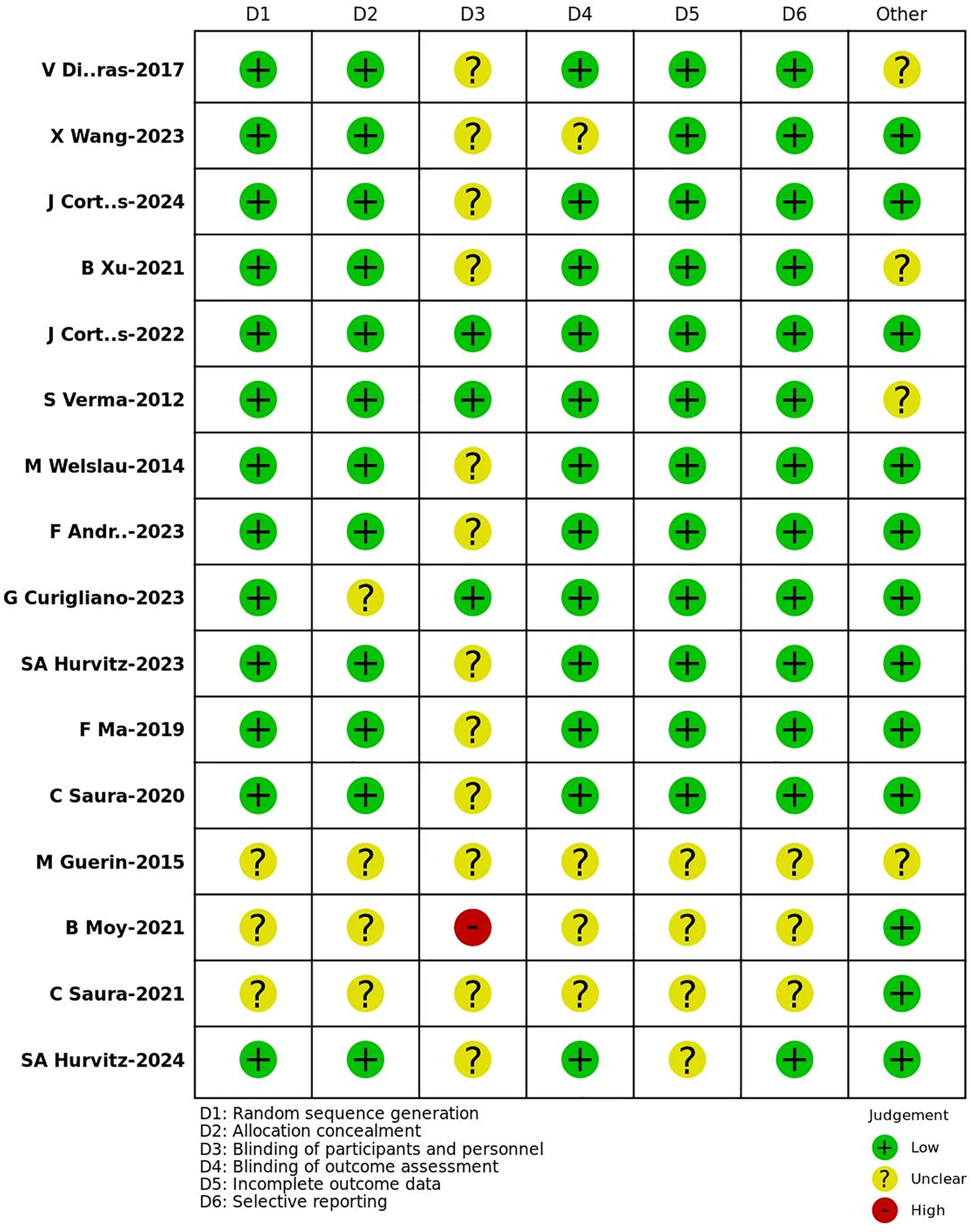
Risk-of-bias assessment for individual trial reports. Risk-of-bias judgments for each included report, grouped by trial family, across seven Cochrane RoB 1 domains: random sequence generation (D1), allocation concealment (D2), blinding of participants and personnel (D3), blinding of outcome assessment (D4), incomplete outcome data (D5), selective reporting (D6), and other bias. Green indicates low risk, yellow indicates unclear risk, and red indicates high risk.

### Geometry of the network

The outcome-specific networks differed in size and connectivity. PFS and OS each formed a connected five-node, four-edge tree; therefore, comparisons between nonadjacent nodes were indirect and node splitting was not estimable. The eligible tucatinib comparison formed a disconnected component for PFS and OS and was not included in their common ranking. Confirmed ORR included five interventions and four direct comparison types; SAEs included five interventions and four direct comparison types; DoR included three interventions and two direct comparison types; any-grade AEs included four interventions and three direct comparison types; ILD/pneumonitis and LVD each included three interventions and two direct comparison types. These quantitative secondary networks were tree-shaped. TTP did not form an eligible quantitative network and was summarized descriptively.

### Results by outcome

#### Progression-free survival.

Six independent randomized trial families comprising 2,731 unique randomized participants contributed to the PFS network. Five of these trial families also contributed to the OS network, whereas PHOEBE contributed only to PFS. The HRs for Lap-Cap, Ner-Cap, PTN-Cap, and T-DM1 versus T-DXd were 5.86 (95% CrI 2.94–11.21), 4.45 (1.53–12.32), 2.20 (0.87–5.36), and 3.30 (2.06–5.28), respectively. The HRs for Lap-Cap versus PTN-Cap and Lap-Cap versus T-DM1 were 2.66 (1.44–4.92) and 1.77 (1.08–2.80), respectively. Network heterogeneity diagnostics were not emphasized because the PFS network had only two residual degrees of freedom for heterogeneity estimation. The DIC was 17.50 for the consistency model and 17.49 for the UME model; because the network contained no closed loop, node splitting was not estimable. Exploratory study-level subgroup analyses were less precise in several strata. In second-line-only trials, the HR for Lap-Cap versus PTN-Cap was 2.66 (1.23–5.80), whereas in mixed second- and later-line trials the HR for T-DM1 versus T-DXd was 3.31 (1.88–5.82). The identical Lap-Cap versus PTN-Cap point estimate in the overall and second-line-only analyses arose because all trials contributing to the PTN-Cap comparison were second-line-only; the different CrIs reflect estimation within the subgroup-specific network. For T-DM1 versus T-DXd, the HR was 3.44 (1.48–7.96) in trials with a high prevalence of baseline brain metastases and 3.02 (0.74–12.36) in trials with rare or unreported brain metastases; the corresponding estimates were 3.03 (0.74–12.18) with high prior pertuzumab exposure and 3.43 (2.32–5.16) with low or absent exposure. In multiregional or Western-predominant trials, the T-DM1 versus T-DXd HR was 3.30 (1.84–5.92); T-DXd was not represented in the Asia-dominant PFS network. SUCRA rankings were T-DXd (99%)> PTN-Cap (71%) > T-DM1 (47%)> Ner-Cap (27%)> Lap-Cap (5%) (Fig 4). Accordingly, the PFS ranking of T-DXd primarily reflects mixed-line, non-Asia-dominant trial evidence and does not directly inform its relative ranking in Asia-dominant populations. Rankings were interpreted together with the HRs and CrIs, and comparisons whose CrIs included 1 were considered imprecise.

**Fig 4:**
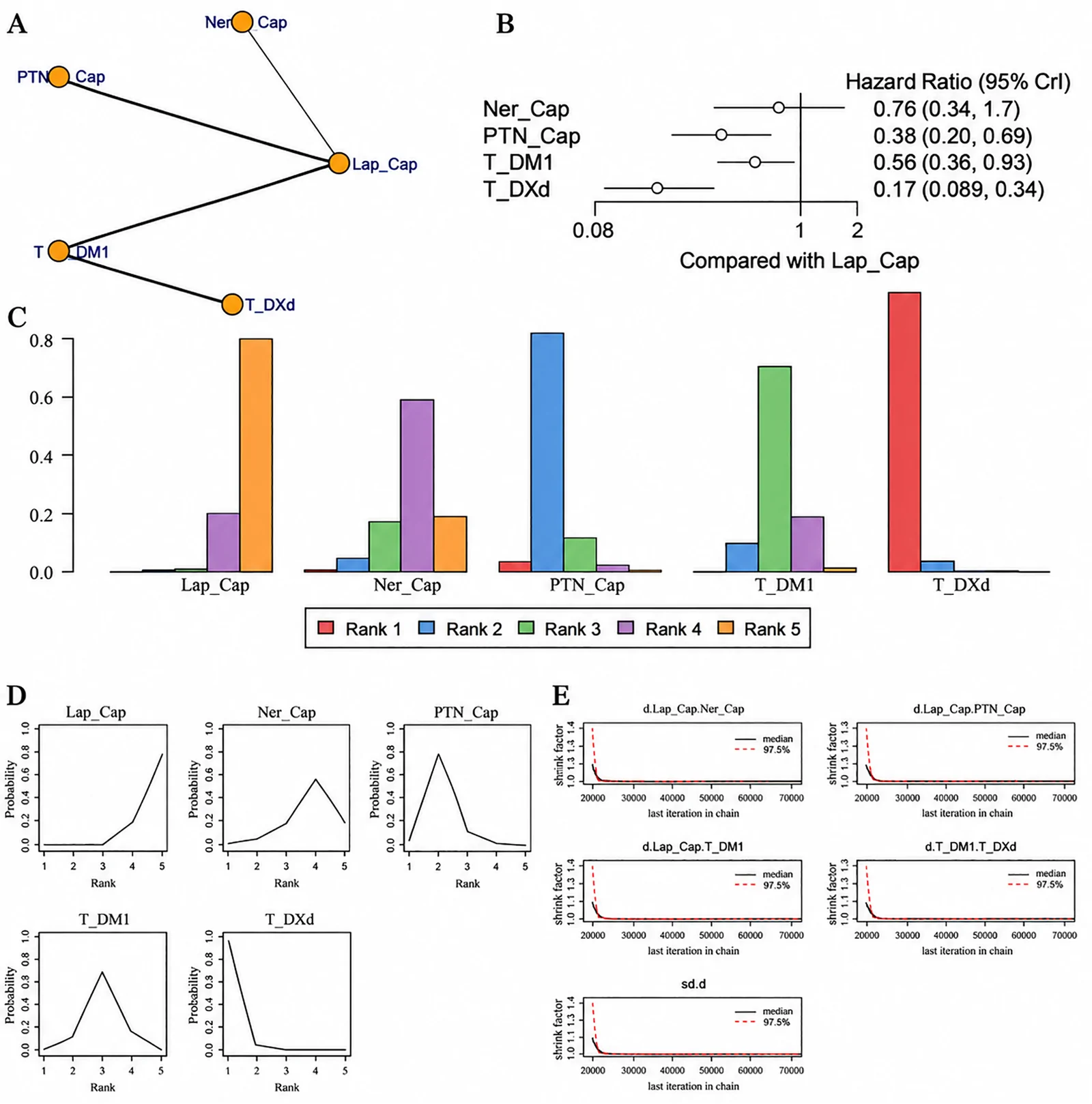
Network meta-analysis results for progression-free survival (PFS). (A) Network plot of treatment comparisons; edge thickness represents the number of independent randomized trials, and node size represents the number of unique randomized participants. (B) Hazard ratios with 95% CrIs for each intervention compared with Lap-Cap; HR < 1 favors the intervention. (C) Rank probability distributions of all interventions. (D) Rank probability curves for individual interventions. (E) Trace plots and Brooks-Gelman-Rubin shrink-factor diagnostics assessing model convergence.

### Overall survival

Five independent randomized trial families comprising 2,464 unique randomized participants contributed to the OS network. All five trial families also contributed to the PFS network, although different publications were used to extract the outcome-specific PFS and OS estimates. The HRs for Lap-Cap, Ner-Cap, PTN-Cap, and T-DM1 versus T-DXd were 2.06 (95% CrI 1.48–2.87), 1.82 (1.09–3.01), 1.41 (0.70–2.91), and 1.52 (1.19–2.01), respectively. The HR for Lap-Cap versus T-DM1 was 1.36 (1.08–1.64). Network heterogeneity diagnostics were not emphasized because the OS network had only one residual degree of freedom for heterogeneity estimation. The DIC was 14.90 for the consistency model and 14.81 for the UME model (difference 0.08); because the network contained no closed loop, node splitting was not estimable. Exploratory study-level subgroup analyses were imprecise in several strata. In mixed second- and later-line trials, the HR for T-DM1 versus T-DXd was 1.52 (1.18–2.00); T-DXd was not represented in the second-line-only OS network. For T-DM1 versus T-DXd, the HR was 1.52 (0.92–2.64) in trials with a high prevalence of baseline brain metastases and 1.56 (0.98–2.49) in trials with rare or unreported brain metastases; the corresponding estimates were 1.56 (0.84–2.90) with high prior pertuzumab exposure and 1.51 (1.00–2.39) with low or absent exposure. In multiregional or Western-predominant trials, the T-DM1 versus T-DXd HR was 1.51 (1.20–1.96). T-DXd was not represented in the Asia-dominant OS network, and the T-DM1 versus Lap-Cap estimate in that stratum was uncertain (HR 1.08, 95% CrI 0.58–2.02), indicating that the apparent treatment hierarchy was not stable across geographic strata. SUCRA rankings were T-DXd (96%)> PTN-Cap (60%) > T-DM1 (56%)> Ner-Cap (30%)> Lap-Cap (8%) (Fig 5). Because T-DXd was absent from both the second-line-only and Asia-dominant OS networks, its OS ranking primarily reflects mixed-line, non-Asia-dominant trial evidence, and its relative position within these subgroups remains uncertain. Rankings were interpreted together with the HRs and CrIs, and comparisons whose CrIs included 1 were considered imprecise.

**Fig 5:**
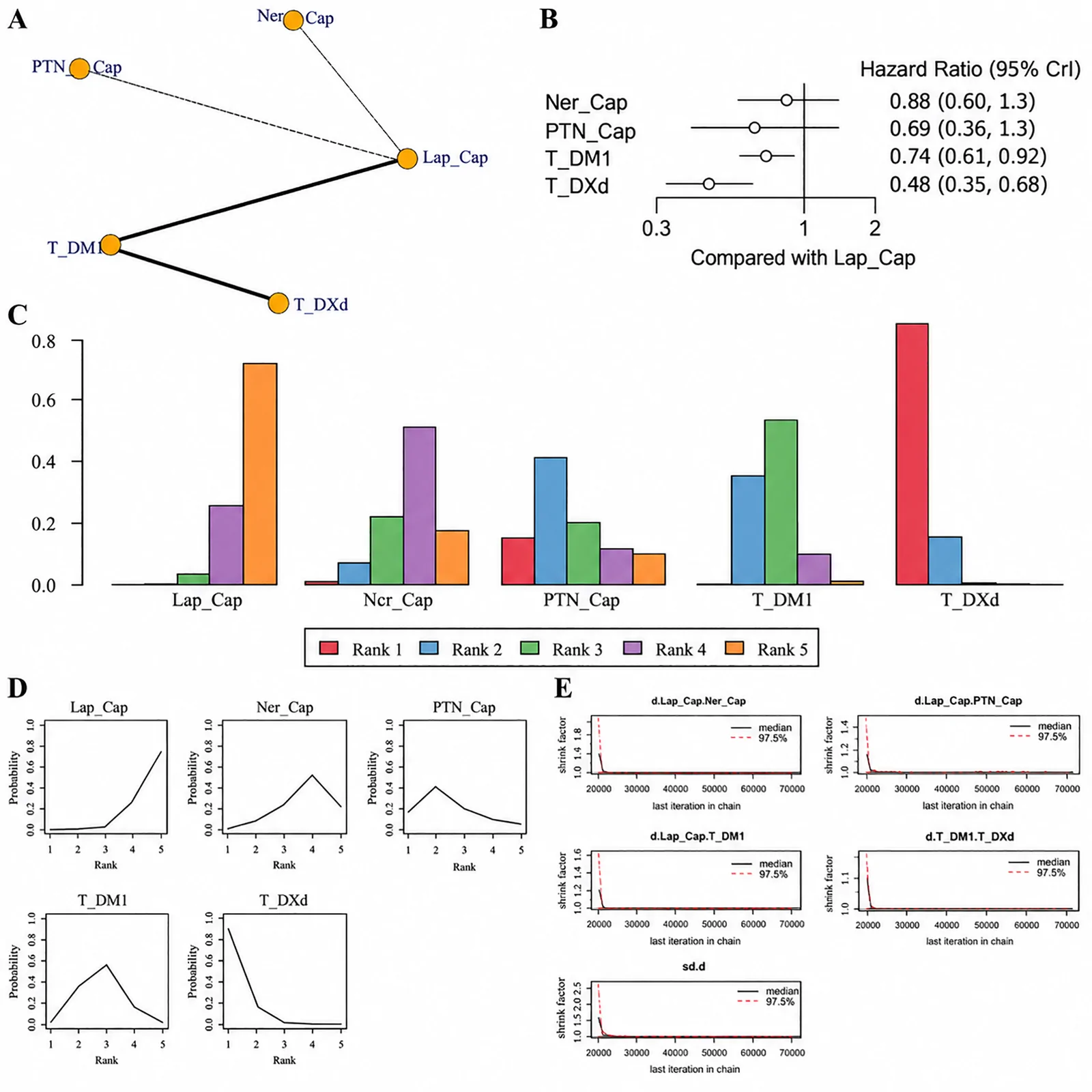
Network meta-analysis results for overall survival (OS). (A) Network plot of treatment comparisons; edge thickness represents the number of independent randomized trials, and node size represents the number of unique randomized participants. (B) Hazard ratios with 95% CrIs for each intervention compared with Lap-Cap; HR < 1 favors the intervention. (C) Rank probability distributions of all interventions. (D) Rank probability curves for individual interventions. (E) Trace plots and Brooks-Gelman-Rubin shrink-factor diagnostics assessing model convergence.

### Confirmed objective response rate

The confirmed ORR network comprised five interventions and four direct comparison types. Using Lap-Cap as the reference, the ORs were 1.35 (95% CrI 0.55–3.32) for Ner-Cap, 2.21 (1.11–4.58) for PTN-Cap, 1.18 (0.64–1.96) for T-DM1, and 7.43 (3.80–13.16) for T-DXd (S3 Fig, panel B). Additional network comparisons showed higher confirmed ORR with T-DXd than with Ner-Cap (OR 5.55, 1.75–15.79), PTN-Cap (OR 3.35, 1.24–8.19), and T-DM1 (OR 6.31, 3.98–10.32). Network heterogeneity diagnostics were not emphasized because the ORR network had only two residual degrees of freedom; the consistency and UME model fits were similar (DIC 34.33 vs 34.26). Because the network was tree-shaped and contained no closed loop, node splitting was not estimable; the DIC comparison was interpreted descriptively rather than as evidence of consistency. SUCRA values were T-DXd 99.46%, PTN-Cap 69.66%, Ner-Cap 39.72%, T-DM1 29.44%, and Lap-Cap 11.72%.

### Duration of response

The exploratory DoR network included three interventions and two direct comparison types from two randomized trial families: EMILIA (T-DM1 vs Lap-Cap) and NALA (Ner-Cap vs Lap-Cap). PHOEBE and DESTINY-Breast03 were not converted because an upper 95% CI limit for DoR was not reached or not estimable. Using Lap-Cap as the reference, the exploratory fixed-effect Bayesian MD was 6.08 months (95% CrI −0.09 to 12.44) for T-DM1 and 2.90 months (−0.11 to 5.93) for Ner-Cap (S4 Fig, panel B). The indirect T-DM1 versus Ner-Cap contrast was 3.19 months (−3.70 to 10.20). All 95% CrIs included zero. Because each direct comparison was informed by a single trial family, between-study heterogeneity was not estimated; the network contained no closed loop, so node splitting was not applicable. Rank probabilities were descriptive and were not used to claim superiority.

### Time to progression

Available TTP evidence did not support quantitative network meta-analysis. PHOEBE reported median TTP of 12.5 months with PTN-Cap and 6.8 months with Lap-Cap, but the upper 95% confidence limit for PTN-Cap was not reached; therefore, the prespecified median/CI-to-mean/SD conversion could not be applied. The Welslau EMILIA report provided FACT-B time to symptom worsening rather than TTP and was excluded as a clinically distinct endpoint. Consequently, no TTP study met the quantitative conversion criteria, and no network MD, 95% interval, heterogeneity estimate, DIC/UME comparison, node-splitting result, or SUCRA/rank probability was generated (S5 Fig).

### Adverse events

The any-grade AE network comprised four interventions and three direct comparison types. Event occurrence was coded as the event; therefore, OR < 1 indicated fewer any-grade adverse events. Direct evidence supported lower AE odds with T-DM1 than with Lap-Cap (OR 0.61, 95% CrI 0.48–0.77). In DESTINY-Breast03, any-grade AEs were more frequent with T-DXd than with T-DM1 (crude OR 12.34). Using Lap-Cap as the reference, the network estimates were OR 0.83 (95% CrI 0.38–1.82) for Ner-Cap, 0.54 (0.26–1.14) for T-DM1, and 6.69 (0.76–59.11) for T-DXd (S6 Fig, panel B). The indirect T-DXd versus Lap-Cap and T-DXd versus Ner-Cap comparisons were highly imprecise, and the any-grade AE ranking was interpreted cautiously because the network was tree-shaped and the DESTINY-Breast03 comparison contained very few non-events. Rank probabilities favored T-DM1 as the lowest-risk regimen for any-grade AEs, whereas T-DXd had the least favorable ranking.

### Serious adverse events

The SAE network comprised five interventions and four direct comparison types. Event occurrence was coded as the event; therefore, OR < 1 indicated fewer serious adverse events. Fixed-effect Bayesian network estimates versus Lap-Cap were OR 1.20 (95% CrI 0.86–1.69) for Ner-Cap, 1.24 (0.55–2.83) for PTN-Cap, 0.90 (0.67–1.21) for T-DM1, and 1.17 (0.71–1.92) for T-DXd (S7 Fig, panel B). The T-DXd versus T-DM1 estimate was OR 1.30 (0.87–1.93), and the indirect T-DXd versus PTN-Cap estimate was 0.94 (0.36–2.45). No comparative CrI excluded 1. T-DM1 had the highest probability of the lowest SAE risk, but ranking was interpreted cautiously because the tree-shaped network was sparse and heterogeneity was not estimated under the fixed-effect model.

### Interstitial lung disease or pneumonitis

The ILD/pneumonitis network comprised two independent randomized trials (n = 718), three interventions, and two direct comparison types: T-DM1 versus Lap-Cap in ELAINA and T-DXd versus T-DM1 in DESTINY-Breast03. Because this two-trial tree contained a zero-event arm and did not support reliable random-effects heterogeneity estimation, the prespecified sparse-network fixed-effect Bayesian model was used. Relative to Lap-Cap, the OR was 0.29 (95% CrI 0.03–2.43) for T-DM1 and 1.62 (0.17–14.45) for T-DXd (S8 Fig, panel B); both comparisons were highly imprecise. By contrast, T-DXd had higher odds of ILD/pneumonitis than T-DM1 (OR 5.55, 95% CrI 2.79–12.16). The network contained no closed loop, so node splitting was not estimable. Treatment ranking was not emphasized because only two trials contributed.

### Left ventricular dysfunction

The LVD network comprised three interventions and two direct comparison types. Event occurrence was coded as the event; therefore, OR < 1 indicated fewer LVD events. Using Lap-Cap as the reference, the network estimates were OR 1.05 (95% CrI 0.40–2.79) for T-DM1 and 3.02 (0.67–13.72) for T-DXd (S9 Fig, panel B). The direct DESTINY-Breast03 comparison yielded a crude OR of 2.87 (95% CI 0.90–9.14) for T-DXd versus T-DM1, indicating numerically more LVD events with T-DXd, although the interval included the null. None of the 95% CrIs excluded 1. Network heterogeneity diagnostics were interpreted cautiously because the LVD network had only one residual degree of freedom and sparse events, precluding a definitive ranking. T-DXd had the least favorable rank probabilities, and panel E of S9 Fig presents the Gelman-Rubin shrink-factor plots used to assess model convergence.

### Publication bias and sensitivity analysis

All quantitative outcome networks contained fewer than 10 independent studies. Accordingly, comparison-adjusted Egger tests were not used for inferential assessment, and no P-value-based inference regarding funnel-plot asymmetry was made. Sensitivity analyses were conducted only when sufficient data and network connectivity were available. Leave-one-trial-family-out analysis was not informative for DoR because removal of either contributing family eliminated one of the two direct comparisons; ILD/pneumonitis was too sparse for informative leave-one-out analysis, and TTP had no quantitative synthesis. For other estimable outcomes, sensitivity analyses examined heterogeneity under the prespecified exclusions or alternative continuity-correction assumptions, with interpretation based on the resulting effect estimates and uncertainty.

### GRADE assessment

Comparison-level certainty ranged from high to very low (S2-S10 Tables in S2 Appendix). Certainty was high for the precise direct T-DM1 versus T-DXd PFS and OS comparisons. Indirect comparisons were generally rated moderate when precise and low when both indirectness and imprecision were present. For DoR, the direct T-DM1 versus Lap-Cap and Ner-Cap versus Lap-Cap comparisons were rated low because of imprecision and the exploratory median-to-mean conversion; the indirect T-DM1 versus Ner-Cap comparison was rated very low. No quantitative GRADE rating was assigned for TTP. For SAEs, T-DM1 versus Lap-Cap and T-DXd versus T-DM1 were rated moderate because direct randomized evidence remained imprecise, whereas the indirect T-DXd versus PTN-Cap comparison was rated low. LVD comparisons were rated low or very low because events were sparse and intervals were wide. The T-DXd versus T-DM1 ILD/pneumonitis comparison was rated moderate because it was direct but rare-event evidence; the Lap-Cap ILD comparisons were rated very low because sparse or zero events produced severe imprecision. No comparison was downgraded solely on the basis of an Egger P value because all networks were below the small-study-effect threshold.

## Discussion

This systematic review and NMA synthesized 16 reports from seven independent randomized trial families of HER2-targeted therapies used after trastuzumab-based treatment. Across efficacy outcomes, T-DXd showed a favorable PFS and OS profile and the highest confirmed ORR. SAE comparisons did not demonstrate a clear difference between regimens, any-grade AE estimates did not indicate a safety advantage for T-DXd, and T-DXd had substantially higher odds of ILD/pneumonitis than T-DM1. DoR and LVD comparisons remained inconclusive, whereas the available TTP data did not support quantitative synthesis. These findings should be interpreted in the context of network connectivity, sparse events, endpoint comparability, and clinical transitivity.

The direct benefit of T-DXd versus T-DM1 is already established by DESTINY-Breast03 [6,20]. The added value of the present NMA is the relative positioning of T-DXd and the other connected treatments for comparisons without head-to-head randomized evidence, including indirect comparisons with Lap-Cap, Ner-Cap, and PTN-Cap. The CrIs for Lap-Cap, Ner-Cap, and T-DM1 versus T-DXd excluded 1 for both PFS and OS, whereas the PTN-Cap versus T-DXd comparisons were imprecise. These indirect estimates depend on the clinical transitivity assumption across studies conducted in different treatment eras and populations. The favorable survival profile of T-DXd may be related to its high drug-to-antibody ratio, bystander activity, and topoisomerase I inhibitor payload, which may enable durable disease control even with heterogeneous or reduced HER2 expression [21–23]. In contrast, TKIs that block HER2 signaling may be more susceptible to resistance mechanisms, potentially limiting long-term benefit. Tucatinib was not included in the connected PFS or OS network because its eligible comparison formed a disconnected component; accordingly, no quantitative survival ranking for tucatinib is inferred from this NMA.

Safety analyses showed no clear SAE advantage for any regimen [24]. The fixed-effect Bayesian estimates were OR 0.90 (95% CrI 0.67–1.21) for T-DM1 versus Lap-Cap and OR 1.30 (0.87–1.93) for T-DXd versus T-DM1. For any-grade AEs, estimates likewise did not support lower odds with T-DXd; direct evidence from DESTINY-Breast03 showed more any-grade AEs with T-DXd than with T-DM1. These findings remain compatible with the broader tolerability advantages reported for T-DM1 in EMILIA [20], but SAE-specific network rankings should not be overstated.

Confirmed ORR was highest with T-DXd: the odds of response were higher than with Lap-Cap, Ner-Cap, PTN-Cap, and T-DM1. PTN-Cap also improved ORR versus Lap-Cap. For DoR, the exploratory analysis was restricted to EMILIA and NALA: T-DM1 and Ner-Cap were numerically longer than Lap-Cap, but both 95% CrIs included zero. DoR for PTN-Cap and T-DXd was not included in the quantitative synthesis because complete two-sided 95% CI summaries were unavailable for the prespecified conversion. Thus, response probability and response durability should not be treated as interchangeable endpoints. TKI-based regimens such as pyrotinib-capecitabine can still achieve clinically meaningful responses, but comparative durability remains uncertain [5,25,26]. The high ORR observed with T-DXd is biologically plausible for an ADC with a potent topoisomerase I inhibitor payload and bystander activity; however, the limited DoR evidence constrains conclusions about longer-term disease control from this secondary outcome alone [27,28].

The ILD/pneumonitis analysis showed higher odds with T-DXd than with T-DM1 (OR 5.55, 95% CrI 2.79–12.16), consistent with broader phase III evidence that ILD is a clinically important T-DXd toxicity [29–31]. Comparisons involving Lap-Cap were imprecise because only ELAINA linked Lap-Cap to the network and its Lap-Cap arm had zero events; the corresponding ORs were 0.29 (0.03–2.43) for T-DM1 versus Lap-Cap and 1.62 (0.17–14.45) for T-DXd versus Lap-Cap. For LVD, the direct comparison suggested numerically more events with T-DXd than with T-DM1, but no comparison was conclusive because event counts were low and intervals were wide. These findings reinforce the need for pulmonary and cardiac surveillance, especially during T-DXd treatment.

Heterogeneity was estimated only where the network contained residual degrees of freedom, and the resulting diagnostics were interpreted cautiously given the small network sizes. For DoR, between-study I² was not meaningfully estimable because the two contributing trial families supplied different direct comparisons, with one study per comparison. The confirmed ORR network was tree-shaped and contained no closed loop; the DoR network was likewise a three-node, two-edge tree. Local inconsistency therefore could not be evaluated by node splitting in either network. The other secondary networks were also predominantly tree-shaped, so local inconsistency was not estimable for those networks. Model-fit statistics do not establish clinical transitivity, particularly across trials differing in treatment line, prior pertuzumab exposure, brain-metastasis prevalence, and treatment era.

A key strength of this study is its outcome-specific comparative framework, which evaluates survival, response, response duration, and clinically relevant toxicities within the networks supported by the available randomized evidence [32,33]. Binary outcomes were defined consistently, with confirmed response treated as the event for ORR and toxicity occurrence treated as the event for safety outcomes, providing a uniform direction for interpretation of ORs. Effect estimates and 95% CrIs, rather than SUCRA rankings alone, were used to draw conclusions. Important uncertainty remains: several survival comparisons were indirect and most secondary networks were sparse. Small-study effects could not be meaningfully assessed because every quantitative network contained fewer than 10 independent studies. The GRADE assessment distinguishes the certainty of direct, indirect, and network evidence and identifies indirectness, imprecision, and sparse events as principal limitations.

The clinical interpretation is a benefit-risk trade-off rather than a single universal hierarchy. Within the connected survival network, T-DXd showed a favorable PFS and OS profile and the highest confirmed ORR. Its efficacy must be balanced against the absence of a clear any-grade AE advantage and against substantially higher ILD/pneumonitis odds than T-DM1. SAE comparisons did not show a clear difference between T-DM1 and Lap-Cap or between T-DXd and T-DM1, so SAE ranking probabilities should be interpreted cautiously [34]. PTN-Cap improved ORR versus Lap-Cap but did not show a clear SAE advantage [35]. Tucatinib-trastuzumab-capecitabine remains clinically important, particularly for central nervous system disease, but its eligible comparison was disconnected from the PFS and OS networks and therefore was not quantitatively ranked here. Geographic subgroup findings also warrant caution: T-DXd was absent from the Asia-dominant networks, and the Asia-dominant T-DM1 versus Lap-Cap OS estimate crossed the null. This pattern may reflect treatment era, prior therapy, or trial mix rather than true geographic effect modification. All subgroup analyses were trial-level and exploratory. Future head-to-head and real-world studies should prioritize CNS outcomes, long-term pulmonary safety, quality of life, and predictive biomarkers for ILD.

Several limitations should be acknowledged. First, aggregate trial-level data prevented adjustment for individual prognostic factors. Second, the connected PFS and OS networks combined true second-line and mixed second- and later-line populations and differed in region, prior pertuzumab exposure, baseline brain-metastasis prevalence, treatment era, and follow-up; clinical transitivity therefore remains an assumption. Third, the tree-shaped survival networks contained no closed loops, and several comparisons were entirely indirect. Fourth, DoR was informed by few trials, TTP could not be quantitatively synthesized because the available summaries did not satisfy the prespecified conversion and endpoint-equivalence criteria, and ILD and LVD analyses were affected by sparse or zero events. Fifth, small-study effects could not be meaningfully assessed because all quantitative networks contained fewer than 10 independent studies; ILD/pneumonitis was especially sparse, and TTP had no quantitative synthesis to assess. Sixth, the tucatinib comparison was disconnected from the common PFS and OS network. Finally, outcome definitions and ascertainment intensity varied across trials, particularly for organ-specific toxicities. Individual-patient-data meta-analysis and standardized prospective safety reporting are needed to reduce these uncertainties.

## Conclusions

This NMA provides a comparative synthesis of HER2-targeted therapies after trastuzumab-based treatment. Within the connected survival network, T-DXd showed a favorable PFS and OS profile and the highest confirmed ORR. Any-grade AE estimates did not indicate a clear safety advantage for T-DXd, and T-DXd had substantially higher odds of ILD/pneumonitis than T-DM1. SAE comparisons did not demonstrate a clear difference between T-DM1 and Lap-Cap or between T-DM1 and T-DXd. DoR and LVD comparisons remained inconclusive, and the available TTP data did not support quantitative synthesis. Because several survival comparisons were indirect and several safety networks were sparse, treatment decisions should be based on effect estimates and 95% CrIs, certainty of evidence, pulmonary and cardiac risk, CNS disease, prior therapy, and patient preferences rather than SUCRA rankings alone.

## Supporting information

S1 Fig Subgroup analyses of PFS from the network meta-analysis. (A) Forest plot of PFS stratified by trials with a higher prevalence of baseline brain metastases (≥15%). (B) Forest plot of PFS stratified by trials with rare or unreported baseline brain metastases. (C) Forest plot of PFS stratified by second-line-only trials. (D) Forest plot of PFS stratified by second- and later-line trials. (E) Forest plot of PFS stratified by trials with low or absent prior pertuzumab exposure. (F) Forest plot of PFS stratified by trials with high prior pertuzumab exposure (≥50–60%). (G) Forest plot of PFS stratified by Asia-dominant trials. (H) Forest plot of PFS stratified by multiregional or Western-predominant trials.
(TIF)

S2 Fig Subgroup analyses of OS from the network meta-analysis. (A) Forest plot of OS stratified by trials with a higher prevalence of baseline brain metastases (≥15%). (B) Forest plot of OS stratified by trials with rare or unreported baseline brain metastases. (C) Forest plot of OS stratified by second-line-only trials. (D) Forest plot of OS stratified by second- and later-line trials. (E) Forest plot of OS stratified by trials with low or absent prior pertuzumab exposure. (F) Forest plot of OS stratified by trials with high prior pertuzumab exposure (≥50–60%). (G) Forest plot of OS stratified by Asia-dominant trials. (H) Forest plot of OS stratified by multiregional or Western-predominant trials.
(TIF)

S3 Fig Network meta-analysis results for confirmed objective response rate. (A) Network plot. (B) Odds ratios with 95% CrIs relative to Lap-Cap; OR > 1 indicates a higher confirmed response probability. (C) Rank probability distributions. (D) Rank probability curves. (E) Convergence diagnostics. The five-node/four-edge network was tree-shaped, so node splitting was not estimable.
(TIF)

S4 Fig Exploratory Bayesian analysis of duration of response. (A) Network plot of the eligible EMILIA and NALA comparisons. (B) Mean differences with 95% CrIs relative to Lap-Cap; positive values indicate longer DoR. (C) Rank probability distributions. (D) Rank probability curves. (E) Convergence diagnostics. The three-node/two-edge network had no closed loop, so node splitting was not applicable.
(TIF)

S5 Fig Assessment of time to progression evidence. (A) TTP-related comparisons identified from the eligible reports. PHOEBE could not be converted because the PTN-Cap upper 95% confidence limit was not reached, whereas the EMILIA/Welslau FACT-B time-to-symptom-worsening endpoint was clinically distinct from TTP and was excluded. (B) Reported PHOEBE medians are shown descriptively and were not converted to a mean difference.
(TIF)

S6 Fig Network meta-analysis results for any-grade adverse events. (A) Network plot. (B) Odds ratios with 95% CrIs; OR < 1 indicates fewer any-grade AEs relative to Lap-Cap. (C) Rank probability distributions. (D) Rank probability curves. (E) Event-coding note summarizing the definition of adverse-event occurrence and the DESTINY-Breast03 direct comparison.
(TIF)

S7 Fig Fixed-effect Bayesian network meta-analysis of serious adverse events. (A) Network plot. (B) Odds ratios with 95% CrIs; OR < 1 indicates fewer SAEs relative to Lap-Cap. (C) Rank probability distributions. (D) Rank probability curves. (E) Brooks-Gelman-Rubin shrink-factor diagnostics for treatment effects. The fixed-effect model was used because the tree-shaped network provided only one residual degree of freedom for heterogeneity.
(TIF)

S8 Fig Fixed-effect Bayesian network meta-analysis of interstitial lung disease or pneumonitis. (A) Network plot. (B) Odds ratios with 95% CrIs; OR < 1 indicates fewer ILD/pneumonitis events relative to Lap-Cap. (C) Rank probability distributions. (D) Rank probability curves for the three treatments in the network. (E) Brooks-Gelman-Rubin shrink-factor diagnostics for the three treatment-effect parameters. The sparse-network fixed-effect model was used because only two trials contributed and one arm had zero events.
(TIF)

S9 Fig Network meta-analysis results for left ventricular dysfunction. (A) Network plot. (B) Odds ratios with 95% CrIs; OR < 1 indicates fewer LVD events relative to Lap-Cap. (C) Rank probability distributions. (D) Rank probability curves for the three treatments in the network. (E) Gelman-Rubin shrink-factor plots for the three treatment-effect parameters used to assess model convergence.
(TIF)

S1 Checklist PRISMA 2020 checklist. (DOCX)

S2 Checklist PRISMA-NMA extension checklist. (DOCX)

S1 Appendix Complete search strategies for PubMed, Embase, Web of Science Core Collection, and CENTRAL, updated July 24, 2026. (DOCX)

S2 Appendix Supplementary Tables S1-S10, including trial-family/report mapping, risk-of-bias judgments, and comparison-level GRADE profiles. (DOCX)

S3 Appendix Supplementary data, analytic inputs, analysis code and outputs, figure map, and file manifest. (ZIP)
