# Supplementary material for: Efficacy and safety of T-DXd versus Other HER2-targeted therapies in second-line and later settings for HER2-positive metastatic breast cancer: A Bayesian network meta-analysis
Source: PLoS One. 2026 Sep 29;21(9):e0358540. doi: 10.1371/journal.pone.0358540 (PMC13623098; doi:10.1371/journal.pone.0358540)
Supplement: Supplementary file 3 [file pone.0358540.s012.docx]

1. PubMed (NCBI)

| **No.** | **Search strategy** | **Result** |
| --- | --- | --- |
| #1 | ("Breast Neoplasms"[Mesh] OR breast cancer*[tiab] OR breast carcinoma*[tiab] OR breast neoplasm*[tiab] OR breast tumor*[tiab] OR breast tumour*[tiab] OR mammary cancer*[tiab] OR mammary carcinoma*[tiab] OR mammary neoplasm*[tiab]) | 535,539 |
| #2 | ("HER2-positive"[tiab] OR "HER2 positive"[tiab] OR "HER-2-positive"[tiab] OR "HER-2 positive"[tiab] OR "ERBB2-positive"[tiab] OR "ERBB2 positive"[tiab] OR "ErbB-2-positive"[tiab] OR "ErbB-2 positive"[tiab] OR (("Receptor, ErbB-2"[Mesh] OR HER2[tiab] OR HER-2[tiab] OR ERBB2[tiab] OR ErbB-2[tiab]) AND (overexpress*[tiab] OR amplif*[tiab]))) | 30,214 |
| #3 | ("trastuzumab deruxtecan"[tiab] OR "fam-trastuzumab deruxtecan-nxki"[tiab] OR T-DXd[tiab] OR TDXd[tiab] OR DS-8201*[tiab] OR Enhertu[tiab] OR "Ado-Trastuzumab Emtansine"[Mesh] OR "trastuzumab emtansine"[tiab] OR "ado-trastuzumab emtansine"[tiab] OR T-DM1[tiab] OR TDM1[tiab] OR Kadcyla[tiab] OR tucatinib[tiab] OR ONT-380[tiab] OR ONT380[tiab] OR Tukysa[tiab] OR pyrotinib[tiab] OR SHR-1258[tiab] OR SHR1258[tiab] OR lapatinib[tiab] OR GW572016[tiab] OR GW-572016[tiab] OR Tykerb[tiab] OR Tyverb[tiab] OR neratinib[tiab] OR HKI-272[tiab] OR HKI272[tiab] OR Nerlynx[tiab] OR capecitabine[tiab] OR Xeloda[tiab]) | 15,876 |
| #4 | ((randomized controlled trial[pt] OR controlled clinical trial[pt] OR random*[tiab] OR placebo*[tiab] OR "drug therapy"[sh] OR trial[tiab] OR groups[tiab]) NOT (animals[mh] NOT humans[mh])) | 6,360,778 |
| #5 | #1 AND #2 AND #3 AND #4 | 2,228 |
| Filter Applied | Article Type: Randomized Controlled Trial | 228 |

Limits: no language restriction; search end date July 24, 2026. The randomized controlled trial filter shown in the final row was applied after combining the search sets.

2. Cochrane Central Register of Controlled Trials (CENTRAL; Cochrane Library/Wiley)

| **No.** | **Search strategy** | **Result** |
| --- | --- | --- |
| #1 | MeSH descriptor: [Breast Neoplasms] explode all trees | 21965 |
| #2 | (breast NEAR/3 (cancer* OR neoplasm* OR carcinoma* OR tumor* OR tumour*)):ti,ab,kw | 53490 |
| #3 | (mammar* NEAR/3 (cancer* OR neoplasm* OR carcinoma* OR tumor* OR tumour*)):ti,ab,kw | 256 |
| #4 | #1 OR #2 OR #3 | 53571 |
| #5 | MeSH descriptor: [Erb-b2 Receptor Tyrosine Kinases] explode all trees | 1886 |
| #6 | ((HER2 OR HER-2 OR ERBB2 OR ErbB-2) NEAR/3 (positive OR overexpress* OR amplif*)):ti,ab,kw | 5758 |
| #7 | #5 OR #6 | 6488 |
| #8 | ("trastuzumab deruxtecan" OR "fam-trastuzumab deruxtecan-nxki" OR T-DXd OR TDXd OR DS-8201* OR Enhertu OR "trastuzumab emtansine" OR "ado-trastuzumab emtansine" OR T-DM1 OR TDM1 OR Kadcyla OR tucatinib OR ONT-380 OR ONT380 OR Tukysa OR pyrotinib OR SHR-1258 OR SHR1258 OR lapatinib OR GW572016 OR GW-572016 OR Tykerb OR Tyverb OR neratinib OR HKI-272 OR HKI272 OR Nerlynx OR capecitabine OR Xeloda):ti,ab,kw | 7403 |
| #9 | #4 AND #7 AND #8 | 1521 |
| Limit | Trials | 1519 |

*Limits: source restricted to Trials (CENTRAL); no language restriction; no additional design filter.*

3. Embase (Embase.com; Elsevier)

| **No.** | **Search strategy** | **Result** |
| --- | --- | --- |
| #1 | ('breast cancer'/exp OR (breast NEAR/3 (cancer* OR neoplasm* OR carcinoma* OR tumor* OR tumour*)):ti,ab,kw OR (mammar* NEAR/3 (cancer* OR neoplasm* OR carcinoma* OR tumor* OR tumour*)):ti,ab,kw) | 889,885 |
| #2 | ('human epidermal growth factor receptor 2 positive breast cancer'/exp OR ((her2 OR 'her-2' OR erbb2 OR 'erbb-2') NEAR/3 (positive OR overexpress* OR amplif*)):ti,ab,kw) | 67,837 |
| #3 | ('trastuzumab deruxtecan'/exp OR 'trastuzumab emtansine'/exp OR 'tucatinib'/exp OR 'pyrotinib'/exp OR 'lapatinib'/exp OR 'neratinib'/exp OR 'capecitabine'/exp OR 'fam-trastuzumab deruxtecan-nxki':ti,ab,kw OR 't-dxd':ti,ab,kw OR tdxd:ti,ab,kw OR 'ds-8201*':ti,ab,kw OR enhertu:ti,ab,kw OR 'ado-trastuzumab emtansine':ti,ab,kw OR 't-dm1':ti,ab,kw OR tdm1:ti,ab,kw OR kadcyla:ti,ab,kw OR 'ont-380':ti,ab,kw OR ont380:ti,ab,kw OR tukysa:ti,ab,kw OR 'shr-1258':ti,ab,kw OR shr1258:ti,ab,kw OR gw572016:ti,ab,kw OR 'gw-572016':ti,ab,kw OR tykerb:ti,ab,kw OR tyverb:ti,ab,kw OR 'hki-272':ti,ab,kw OR hki272:ti,ab,kw OR nerlynx:ti,ab,kw OR xeloda:ti,ab,kw) | 71,517 |
| #4 | ('randomized controlled trial'/exp OR 'controlled clinical trial'/exp OR 'randomization'/exp OR 'single blind procedure'/exp OR 'double blind procedure'/exp OR 'crossover procedure'/exp OR random*:ti,ab OR placebo*:ti,ab OR trial:ti) | 3,365,394 |
| #5 | #1 AND #2 AND #3 AND #4 | 2,559 |
| #6 | #5 NOT ('animal'/exp NOT 'human'/exp) | 2,541 |
| Filter Applied | StudyType: Randomized Controlled Trial topic | 217 |

Limits: no language restriction; search end date July 24, 2026. Conference abstracts were retrieved to maximize sensitivity but were excluded at eligibility assessment because the final inclusion criterion required a full-text randomized trial article.

4. Web of Science Core Collection (Clarivate)

| **No.** | **Search strategy** | **Result** |
| --- | --- | --- |
| #1 | TS=((breast NEAR/3 (cancer* OR neoplasm* OR carcinoma* OR tumor* OR tumour*)) OR (mammar* NEAR/3 (cancer* OR neoplasm* OR carcinoma* OR tumor* OR tumour*))) | 739,528 |
| #2 | TS=((HER2 OR HER-2 OR ERBB2 OR ErbB-2) NEAR/3 (positive OR overexpress* OR amplif*)) | 35,092 |
| #3 | TS=("trastuzumab deruxtecan" OR "fam-trastuzumab deruxtecan-nxki" OR T-DXd OR TDXd OR DS-8201* OR Enhertu OR "trastuzumab emtansine" OR "ado-trastuzumab emtansine" OR T-DM1 OR TDM1 OR Kadcyla OR tucatinib OR ONT-380 OR ONT380 OR Tukysa OR pyrotinib OR SHR-1258 OR SHR1258 OR lapatinib OR GW572016 OR GW-572016 OR Tykerb OR Tyverb OR neratinib OR HKI-272 OR HKI272 OR Nerlynx OR capecitabine OR Xeloda) | 26,524 |
| #4 | TS=(random* OR placebo* OR "controlled trial*" OR "clinical trial*" OR "double blind*" OR "single blind*" OR crossover* OR "cross-over") | 3,158,766 |
| #5 | #1 AND #2 AND #3 AND #4 | 1,627 |

Limits: no language restriction; search end date July 24, 2026. Document types were not restricted during retrieval; conference abstracts and proceedings were excluded during study selection.
