# Supplementary material for: Efficacy and safety of T-DXd versus Other HER2-targeted therapies in second-line and later settings for HER2-positive metastatic breast cancer: A Bayesian network meta-analysis
Source: PLoS One. 2026 Sep 29;21(9):e0358540. doi: 10.1371/journal.pone.0358540 (PMC13623098; doi:10.1371/journal.pone.0358540)
Supplement: Supplementary file 4 [file pone.0358540.s013.docx]

**S2 Appendix — Supplementary Tables S1-S10**

This appendix contains the trial-family/report mapping and comparison-level certainty assessments cited in the manuscript. Certainty judgments use the GRADE domains described in the Methods. Ratings are based on the analytic inputs and outputs described in the Methods; treatment ranking alone was not used to increase certainty.

# S1 Table. Included reports, associated trial families, and risk-of-bias judgments.

# Panel A. Trial-report mapping.

| **Trial family** | **Included report identifiers** |
| --- | --- |
| EMILIA | Verma 2012; Welslau 2014; Dieras 2017; Guerin 2015 |
| ELAINA | Wang XJ 2023 |
| DESTINY-Breast03 | Cortes 2022; Curigliano 2023; Hurvitz 2023; Cortes 2024; Hurvitz 2024 |
| PHOEBE | Xu 2021 |
| Phase II pyrotinib | Ma 2019 |
| NALA | Saura 2020; Moy 2021; Saura 2021 |
| DESTINY-Breast02 | Andre 2023 |

The 16 reports represented seven independent randomized trial families. Multiple reports from the same trial family were linked and were not counted as independent trials or added repeatedly when calculating participant totals. For each outcome, the most complete or most recent outcome-specific report was used.

**Panel B. Risk-of-bias judgments for included reports.**

| **Report identifier** | **Random sequence** | **Allocation concealment** | **Participant/personnel blinding** | **Outcome assessment blinding** | **Incomplete data** | **Selective reporting** | **Other bias** |
| --- | --- | --- | --- | --- | --- | --- | --- |
| Dieras 2017 | Low | Low | Unclear | Low | Low | Low | Unclear |
| Wang XJ 2023 | Low | Low | Unclear | Unclear | Low | Low | Low |
| Cortes 2024 | Low | Low | Unclear | Low | Low | Low | Low |
| Xu 2021 | Low | Low | Unclear | Low | Low | Low | Unclear |
| Cortes 2022 | Low | Low | Low | Low | Low | Low | Low |
| Verma 2012 | Low | Low | Low | Low | Low | Low | Unclear |
| Welslau 2014 | Low | Low | Unclear | Low | Low | Low | Low |
| Andre 2023 | Low | Low | Unclear | Low | Low | Low | Low |
| Curigliano 2023 | Low | Unclear | Low | Low | Low | Low | Low |
| Hurvitz 2023 | Low | Low | Unclear | Low | Low | Low | Low |
| Ma 2019 | Low | Low | Unclear | Low | Low | Low | Low |
| Saura 2020 | Low | Low | Unclear | Low | Low | Low | Low |
| Guerin 2015 | Unclear | Unclear | Unclear | Unclear | Unclear | Unclear | Unclear |
| Moy 2021 | Unclear | Unclear | High | Unclear | Unclear | Unclear | Low |
| Saura 2021 | Unclear | Unclear | Unclear | Unclear | Unclear | Unclear | Low |
| Hurvitz 2024 | Low | Low | Unclear | Low | Unclear | Low | Low |

Abbreviations and judgments follow Figures 2 and 3. Report identifiers are shortened author-year labels used throughout the review materials.

# S2 Table. GRADE certainty assessment for progression-free survival.

| **Comparison** | **Network estimate (95% CrI)** | **Evidence source** | **Imprecision** | **Sparse events** | **Certainty** | **Reason for rating** |
| --- | --- | --- | --- | --- | --- | --- |
| Lap-Cap vs T-DXd | HR 5.86 (2.94-11.21) | Indirect | No | No | Moderate | Downgraded one level for indirectness. |
| Ner-Cap vs T-DXd | HR 4.45 (1.53-12.32) | Indirect | Some | No | Low | Downgraded for indirectness and wide CrI. |
| PTN-Cap vs T-DXd | HR 2.20 (0.87-5.36) | Indirect | Yes | No | Low | Downgraded for indirectness and imprecision; CrI includes no effect. |
| T-DM1 vs T-DXd | HR 3.30 (2.06-5.28) | Direct | No | No | High | Precise direct randomized evidence; no serious downgrade identified. |

GRADE categories: high, moderate, low, and very low. Directness indicates whether the comparison included direct randomized evidence, indirect evidence, or both. CrI = credible interval.

# S3 Table. GRADE certainty assessment for overall survival.

| **Comparison** | **Network estimate (95% CrI)** | **Evidence source** | **Imprecision** | **Sparse events** | **Certainty** | **Reason for rating** |
| --- | --- | --- | --- | --- | --- | --- |
| Lap-Cap vs T-DXd | HR 2.06 (1.48-2.87) | Indirect | No | No | Moderate | Downgraded one level for indirectness. |
| Ner-Cap vs T-DXd | HR 1.82 (1.09-3.01) | Indirect | Some | No | Low | Downgraded for indirectness and imprecision. |
| PTN-Cap vs T-DXd | HR 1.41 (0.70-2.91) | Indirect | Yes | No | Low | Downgraded for indirectness and imprecision. |
| T-DM1 vs T-DXd | HR 1.52 (1.19-2.01) | Direct | No | No | High | Precise direct randomized evidence; no serious downgrade identified. |

GRADE categories: high, moderate, low, and very low. Directness indicates whether the comparison included direct randomized evidence, indirect evidence, or both. CrI = credible interval.

# S4 Table. GRADE certainty assessment for confirmed objective response rate.

| **Comparison** | **Network estimate (95% CrI)** | **Evidence source** | **Imprecision** | **Sparse events** | **Certainty** | **Reason for rating** |
| --- | --- | --- | --- | --- | --- | --- |
| PTN-Cap vs Lap-Cap | OR 2.21 (1.11-4.58) | Direct | Some | No | Moderate | Direct evidence; downgraded for limited precision. |
| T-DXd vs Lap-Cap | OR 7.43 (3.80-13.16) | Indirect | No | No | Moderate | Indirect network estimate; downgraded for study-level indirectness across treatment eras. |
| T-DXd vs PTN-Cap | OR 3.35 (1.24-8.19) | Indirect | Some | No | Low | Downgraded for indirectness and wide CrI. |
| T-DXd vs T-DM1 | OR 6.31 (3.98-10.32) | Direct | No | No | High | Precise direct randomized evidence. |

GRADE categories: high, moderate, low, and very low. Directness indicates whether the comparison included direct randomized evidence, indirect evidence, or both. CrI = credible interval.

# S5 Table. GRADE certainty assessment for duration of response. Estimates correspond to the fixed-effect Bayesian DoR analysis based on EMILIA and NALA.

| **Comparison** | Bayesian estimate (95% CrI) | **Evidence source** | **Imprecision** | **Sparse events** | **Certainty** | **Reason for rating** |
| --- | --- | --- | --- | --- | --- | --- |
| Ner-Cap vs Lap-Cap | MD 2.90 months (-0.11 to 5.93) | Direct | Yes | No | Low | Exploratory converted endpoint; CrI includes no difference. |
| T-DM1 vs Lap-Cap | MD 6.08 months (-0.09 to 12.44) | Direct | Yes | No | Low | Exploratory converted endpoint; CrI includes no difference. |
| T-DM1 vs Ner-Cap | MD 3.19 months (-3.70 to 10.20) | Indirect | Yes | No | Very low | Indirect comparison plus exploratory conversion and imprecision. |

GRADE categories: high, moderate, low, and very low. Directness indicates whether the comparison included direct randomized evidence, indirect evidence, or both. CrI = credible interval.

# S6 Table. GRADE status for time to progression. Quantitative GRADE certainty was not assessed because the available TTP evidence did not support a quantitative synthesis. The table records why each candidate comparison was not estimable or not eligible.

| **Comparison** | Quantitative estimate / status | **Evidence source** | **Imprecision** | **Sparse events** | **Certainty** | **Reason for rating** |
| --- | --- | --- | --- | --- | --- | --- |
| PTN-Cap vs Lap-Cap | Not estimable | Direct descriptive source only | Not assessed | N/A | Not assessed | PHOEBE reported median TTP, but the PTN-Cap upper 95% CI was not reached; the prespecified conversion could not be applied. |
| T-DM1 vs Lap-Cap | Not eligible for TTP synthesis | Clinically non-equivalent endpoint | N/A | N/A | Not assessed | The Welslau/EMILIA endpoint was FACT-B time to symptom worsening rather than TTP and was excluded. |

GRADE categories: high, moderate, low, and very low. Directness indicates whether the comparison included direct randomized evidence, indirect evidence, or both. CrI = credible interval.

# S7 Table. GRADE certainty assessment for any-grade adverse events. This table displays the direction-corrected GRADE ratings for AE comparisons, including direct and network estimates of odds ratios and evidence quality.

| **Comparison** | **Network estimate (95% CrI)** | **Evidence source** | **Imprecision** | **Sparse events** | **Certainty** | **Reason for rating** |
| --- | --- | --- | --- | --- | --- | --- |
| Ner-Cap vs Lap-Cap | OR 0.83 (0.38-1.82) | Direct | Yes | No | Low | Direct evidence; CrI includes important benefit and harm. |
| T-DM1 vs Lap-Cap | OR 0.54 (0.26-1.14) | Direct | Yes | No | Low | Direct evidence; CrI includes important benefit and harm. |
| T-DXd vs Lap-Cap | OR 6.69 (0.76-59.11) | Indirect | Yes | Some | Very low | Indirect estimate with sparse non-events and a very wide CrI. |
| T-DXd vs T-DM1 | OR 12.34 (1.59-95.59) | Direct | Yes | Some | Low | Direction corrected; direct estimate indicates more any-grade AEs with T-DXd, but sparse non-events and a wide CrI lower certainty. |

GRADE categories: high, moderate, low, and very low. Directness indicates whether the comparison included direct randomized evidence, indirect evidence, or both. CrI = credible interval.

# S8 Table. GRADE certainty assessment for serious adverse events. Estimates correspond to the final fixed-effect Bayesian SAE network; event occurrence is the event, so OR < 1 indicates fewer SAEs with the first-named treatment.

| **Comparison** | Fixed-effect Bayesian estimate (95% CrI) | **Evidence source** | **Imprecision** | **Sparse events** | **Certainty** | **Reason for rating** |
| --- | --- | --- | --- | --- | --- | --- |
| T-DM1 vs Lap-Cap | OR 0.90 (0.67-1.21) | Direct | Yes | No | Moderate | Direct randomized evidence; downgraded for imprecision because the CrI includes no effect. |
| T-DXd vs PTN-Cap | OR 0.94 (0.36-2.45) | Indirect | Yes | No | Low | Indirect and imprecise; the CrI includes clinically important benefit and harm. |
| T-DXd vs T-DM1 | OR 1.30 (0.87-1.93) | Direct | Yes | No | Moderate | Direct randomized evidence; downgraded for imprecision because the CrI includes no effect and clinically relevant harm. |

GRADE categories: high, moderate, low, and very low. Directness indicates whether the comparison included direct randomized evidence, indirect evidence, or both. CrI = credible interval.

# S9 Table. GRADE certainty assessment for interstitial lung disease or pneumonitis. Estimates correspond to the final sparse-network fixed-effect Bayesian model; the T-DXd versus T-DM1 comparison is direct, whereas Lap-Cap comparisons remain imprecise because of sparse/zero events.

| **Comparison** | Fixed-effect Bayesian estimate (95% CrI) | **Evidence source** | **Imprecision** | **Sparse events** | **Certainty** | **Reason for rating** |
| --- | --- | --- | --- | --- | --- | --- |
| T-DXd vs T-DM1 | OR 5.55 (2.79-12.16) | Direct | No | Yes | Moderate | Direct randomized evidence shows more ILD/pneumonitis with T-DXd; downgraded for rare events. |
| T-DM1 vs Lap-Cap | OR 0.29 (0.03-2.43) | Direct | Yes | Yes | Very low | Zero/sparse events and severe imprecision; the CrI includes substantial benefit and harm. |
| T-DXd vs Lap-Cap | OR 1.62 (0.17-14.45) | Indirect | Yes | Yes | Very low | Indirect, sparse-event evidence with a very wide CrI. |

GRADE categories: high, moderate, low, and very low. Directness indicates whether the comparison included direct randomized evidence, indirect evidence, or both. CrI = credible interval.

# S10 Table. GRADE certainty assessment for left ventricular dysfunction. This table summarizes the direction-corrected GRADE assessments for LVD evidence, with odds ratios, certainty ratings, and notes on sparse-event uncertainty.

| **Comparison** | **Network estimate (95% CrI)** | **Evidence source** | **Imprecision** | **Sparse events** | **Certainty** | **Reason for rating** |
| --- | --- | --- | --- | --- | --- | --- |
| T-DM1 vs Lap-Cap | OR 1.05 (0.40-2.79) | Direct | Yes | Yes | Low | Rare events and a wide CrI that includes benefit and harm. |
| T-DXd vs Lap-Cap | OR 3.02 (0.67-13.72) | Indirect | Yes | Yes | Very low | Indirect sparse-event estimate with a very wide CrI. |
| T-DXd vs T-DM1 | OR 2.87 (0.90-9.14) | Direct | Yes | Yes | Low | Direction-corrected direct estimate suggests numerically more LVD events with T-DXd, but rare events and a wide CrI lower certainty. |

GRADE categories: high, moderate, low, and very low. Directness indicates whether the comparison included direct randomized evidence, indirect evidence, or both. CrI = credible interval.
