# Supplementary material for: Efficacy and safety of T-DXd versus Other HER2-targeted therapies in second-line and later settings for HER2-positive metastatic breast cancer: A Bayesian network meta-analysis
Source: PLoS One. 2026 Sep 29;21(9):e0358540. doi: 10.1371/journal.pone.0358540 (PMC13623098; doi:10.1371/journal.pone.0358540)
Supplement: Supplementary file 6 [file pone.0358540.s016.docx]

## Response to the Academic Editor and Reviewers

| **Manuscript ID** | PONE-D-26-23670 |
| --- | --- |
| **Title** | Efficacy and Safety of T-DXd Versus Other HER2-Targeted Therapies in Second-Line HER2-Positive Metastatic Breast Cancer: A Bayesian Network Meta-Analysis |

**Dear Dr. Shoari,**

We sincerely thank you and the reviewers for the careful and constructive evaluation of our manuscript. We revised the manuscript to strengthen methodological reporting, statistical consistency, and coherence across the main text, Supporting Information, figures, and analysis code. The literature search remains updated through July 24, 2026, and the evidence base comprises 16 reports representing seven independent randomized trial families and 3,339 unique randomized participants. Secondary outcomes were analyzed using consistent endpoint definitions and event coding. Sparse SAE and ILD/pneumonitis networks were evaluated with fixed-effect Bayesian models, DoR was analyzed exploratorily using the prespecified summary-statistic conversion, and TTP was reported descriptively because the available data did not satisfy the criteria for quantitative synthesis. The PRISMA materials, GRADE profiles, figure legends, and S3 data/code package were revised accordingly. All changes are reflected in the marked manuscript. Our point-by-point responses are provided below.

## Academic Editor and Journal Requirements

Journal Requirement 1. Please ensure that the manuscript meets PLOS ONE style requirements, including file naming.

**Response:** We revised the manuscript structure and formatting to conform more closely to PLOS ONE requirements. We prepared clean and marked manuscript files, removed supplementary figure images from the manuscript body, organized Figure 1-5 and Figure S1-S9 as separate figure files, and standardized the Supporting Information file names for the revised submission package.

**Location of revision:** Manuscript structure; Figure Legend file; separately prepared Figure 1-5 and Figure S1-S9 files; S1-S3 Supporting Information materials.

Journal Requirement 2. Funding information should not appear in the manuscript and should be reported only in the online Funding Statement.

**Response:** We removed funding-related disclosure text from the manuscript. The manuscript retains only the CRediT author-contribution role “funding acquisition,” which describes an author contribution and is not a funding disclosure. The funding source will be reported only in the online submission form, as requested.

**Location of revision:** Declarations and Author Contributions sections.

Journal Requirement 3. Please clarify the role of the funders and include the amended Role of Funder statement in the cover letter.

**Response:** We confirm that the funders had no role in the conduct or reporting of the study. The following statement will be included in the cover letter and the online submission record: “The funders had no role in study design, data collection and analysis, decision to publish, or preparation of the manuscript.”

Journal Requirement 4. “All raw data and code are available upon request” does not comply with the PLOS data policy. Data underlying the findings must be publicly available through a repository, the manuscript, or Supporting Information.

**Response:** We agree and have replaced the “available upon request” statement. The revised Data Availability Statement now states that the data and code used in this study are included in S3 Appendix (Supplementary Data and Code).

**Location of revision:** Data Availability declaration; Supporting Information file list; S3 Appendix. Supplementary Data and Code.

**Revised text:** “The data and code used in this study are included in S3 Appendix (Supplementary Data and Code).”

Journal Requirement 5. Supplementary figures should be removed from the manuscript file, uploaded as Supporting Information, and accompanied by legends after the References.

**Response:** We removed all supplementary figure images from the manuscript body and prepared Figure S1-S9 as separate Supporting Information figure files. Complete legends for Figure 1-5 and S1-S9 Figs are provided in the updated Figure Legend file and the S1-S9 Fig legends are also retained after the References in the manuscript.

**Location of revision:** Figure Legend file; Supporting information legends after the References; separate Figure S1-S9 files.

Journal Requirement 6. Reviewer-recommended citations should be evaluated for relevance; there is no requirement to cite them unless directed by the editor.

**Response:** We reviewed the reference list for relevance and consistency. The reviewers did not require citation of specific publications. We added or retained references only where they directly supported the revised methodological and clinical interpretation.

## Reviewer 1

The manuscript is well structured and methodologically sound and is suitable for publication with minor revisions for clarity and formatting.

**Response:** We thank the reviewer for this positive assessment. In response to the request for greater clarity and formatting consistency, we refined the title and abstract, distinguished publication-level reports from independent randomized trial families, clarified outcome-specific network connectivity, standardized statistical terminology and secondary-outcome definitions, expanded the methods and limitations, updated the figure legends, and reorganized the Supporting Information. We believe these changes substantially improve clarity, reproducibility, and presentation.

## Reviewer 2

**General response:** We appreciate the reviewer's detailed assessment. In response to these comments, we repeated the secondary-outcome analyses using uniform endpoint definitions and event coding. This yielded revised safety estimates and showed that the available TTP summaries did not satisfy the prespecified criteria for quantitative synthesis. We therefore revised the manuscript, S2 GRADE tables, figures, and S3 analysis package so that all materials report consistent outcome definitions, model specifications, effect estimates, and interpretations.

## Major Concern 1. Data Availability Statement

The statement “All raw data and code are available upon request” is not compliant. Data, extracted trial information, and R code should be made publicly available with unrestricted access.

**Response:** We agree that the original statement was inadequate and have removed it. The revised submission includes the extracted data, analysis inputs, reproducible R/JAGS scripts, manifests, and analysis outputs in S3 Appendix (Supplementary Data and Code), and the Data Availability Statement has been revised accordingly.

**Location of revision:** Data Availability declaration; Supporting Information file list; S3 Appendix. Supplementary Data and Code.

**Revised text:** “The data and code used in this study are included in S3 Appendix (Supplementary Data and Code).”

## Major Concern 2. Inconsistency Between the Abstract and Main-Text Safety Results

The abstract incorrectly attributed OR 0.65 (95% CrI 0.46-0.96) to T-DXd for serious adverse events; this estimate actually represents T-DM1 versus Lap-Cap. The safety statement was misleading and should be corrected.

**Response:** We agree and thank the reviewer for identifying the original safety attribution error. Following this observation, the secondary safety outcomes were analyzed with toxicity occurrence consistently coded as the event, and the sparse safety networks were re-estimated using the prespecified Bayesian framework. The previously reported SAE estimates of 0.65 and 0.95 were therefore replaced. In the fixed-effect Bayesian SAE network, neither T-DM1 versus Lap-Cap nor T-DXd versus T-DM1 showed a clear difference. The fixed-effect Bayesian ILD/pneumonitis analysis continued to show substantially higher odds with T-DXd than with T-DM1. The Abstract, Results, Discussion, Conclusion, GRADE profiles, Figures S7-S8, and S3 code/output files consistently report these estimates and interpretations.

**Location of revision:** Abstract - Results and Conclusion; Results - any-grade AE, SAE, ILD/pneumonitis, and LVD sections; Discussion; Conclusion; S2 Appendix; Figures S6-S9; S3 Appendix.

**Revised text:** “Any-grade AE estimates did not indicate a safety advantage for T-DXd. SAE comparisons were also inconclusive: T-DM1 vs Lap-Cap OR 0.90 (95% CrI 0.67-1.21) and T-DXd vs T-DM1 OR 1.30 (0.87-1.93). T-DXd had higher ILD/pneumonitis odds than T-DM1 (OR 5.55, 2.79-12.16); LVD differences were unclear.”

## Major Concern 3. PRISMA-NMA Checklist and Flow Diagram

A completed PRISMA-NMA checklist was missing. The flow diagram required a more transparent breakdown of exclusions, particularly the large “wrong intervention” category, and alignment with PRISMA 2020 guidance.

**Response:** We addressed all three points. First, the completed PRISMA 2020 checklist and PRISMA-NMA checklist are now supplied as separate S1 Supporting Information files, and the complete search strategies are supplied as S1 Appendix Search Strategies. Second, after updating the search, we rebuilt the PRISMA 2020 flow diagram using the updated search counts rather than the previous broad “wrong intervention” category. The revised flow reports database-specific yields, duplicate removal, title/abstract exclusions with explicit categories, and full-text exclusions with explicit reasons. Third, the Study Selection text was rewritten to match Figure 1 exactly and distinguishes 16 publication-level reports from seven independent randomized trial families.

**Location of revision:** Methods - Reporting framework and Literature Search; Results - Study Selection; Figure 1; S1 PRISMA-NMA checklist-2020 checklist; S1 PRISMA-NMA checklist-NMA checklist; S1 Appendix Search Strategies.

**Revised text:** “The updated searches identified 3,591 records... After removal of 742 duplicates, 2,849 records were screened... Thirty-two full-text articles were assessed. Sixteen were excluded for five explicit reasons. Sixteen reports representing seven independent randomized trial families and 3,339 unique randomized participants were included.”

## Major Concern 4. Search-Date Discrepancy and Search Strategy

Please clarify whether the search was updated, provide the complete search strategies for all databases, and explain how non-English literature was handled. If no update was performed, the gap should be acknowledged as a limitation.

**Response:** We performed an updated search through July 24, 2026, before resubmission. The update retrieved 3,591 records in total and did not identify an additional eligible randomized trial family. Complete reproducible strategies for PubMed, Embase, Web of Science Core Collection, and CENTRAL are provided in S1 Appendix Search Strategies. No language restrictions were applied, and all 16 included full-text reports were published in English. Conference abstracts and proceedings were captured by the broad searches but were not eligible for inclusion as full-text article reports under the prespecified criteria.

**Location of revision:** Abstract - Methods; Methods - Literature Search; Results - Study Selection; Discussion; Figure 1; S1 Appendix Search Strategies.

**Revised text:** “An updated search was completed on July 24, 2026... No language restrictions were applied, and all 16 included reports were published in English... The evidence base comprised 16 reports representing seven independent randomized trial families.”

## Minor Concern 1. Abstract Length and Structure

The abstract substantially exceeded the PLOS ONE 300-word limit and should be condensed into Background, Methods, Results, and Conclusion.

**Response:** We rewrote the abstract as a structured 300-word abstract excluding the four section headings, meeting the PLOS ONE limit. The revised version reports the report/trial-family structure, primary comparative estimates, secondary efficacy findings, safety results, and a cautious conclusion.

**Location of revision:** Abstract.

## Minor Concern 2. Figure and Table Legends

The legends for Figures 4 and 5 were insufficiently differentiated; supplementary figure legends were truncated or garbled; and the risk-of-bias color coding should be defined explicitly.

**Response:** We updated the standalone Figure Legend file to cover Figure 1-5 and Figure S1-S9 and to correspond with the revised figure set. Figure 4 is explicitly the PFS NMA and Figure 5 the OS NMA. The S3-S9 legends now describe the outcome-specific networks and models directly: exploratory Bayesian analysis for DoR, descriptive reporting of TTP, event-based interpretation of any-grade AE and LVD outcomes, and fixed-effect Bayesian analyses for SAE and ILD/pneumonitis. Figures 2 and 3 define green as low risk, yellow as unclear risk, and red as high risk.

**Location of revision:** Updated Figure Legend file; Supporting information legends after the References; separate Figure 1-5 and Figure S1-S9 files.

**Revised text:** “Green indicates low risk, yellow indicates unclear risk, and red indicates high risk.”

## Minor Concern 3. Statistical Reporting Consistency

Please standardize “95% credible interval/95% CrI,” distinguish frequentist P values from Bayesian inference, and clarify the use of I² in a Bayesian NMA.

**Response:** We standardized “95% CrI” after first definition and separated Bayesian inference from frequentist diagnostics. Bayesian NMA conclusions are based on posterior effect estimates and 95% CrIs. Frequentist Q/I² statistics are used only descriptively for direct evidence when meaningfully estimable. We no longer make a blanket claim of low network heterogeneity. Node splitting is performed only when a closed loop provides both direct and indirect evidence; the ORR and DoR networks are tree-shaped and do not permit node splitting. All quantitative outcome networks contain fewer than 10 independent studies, so comparison-adjusted Egger tests are not treated as valid inferential assessments and no P-value-based publication-bias claim is retained. Sparse SAE and ILD/pneumonitis networks use fixed-effect Bayesian models because heterogeneity is not reliably estimable.

**Location of revision:** Methods - Statistical Analysis, network geometry and convergence; Results - outcome-specific analyses and Publication bias and sensitivity analysis; Discussion - heterogeneity/inconsistency paragraph; S3 Appendix.

**Revised text:** “Bayesian estimates were reported with 95% CrIs. Random-effects Bayesian models were used where between-study heterogeneity could be estimated with reasonable support. The SAE and ILD/pneumonitis networks used fixed-effect Bayesian models because their sparse structures did not support reliable heterogeneity estimation. Comparison-adjusted Egger tests were not treated as inferential assessments when fewer than 10 independent studies contributed.”

## Minor Concern 4. GRADE Assessment Details

The GRADE tables were not accessible, the relationship between direct and network ratings required explanation, and the imprecision criterion should be specified.

**Response:** We provide complete comparison-level GRADE profiles in S2 Appendix and ensure that each rating corresponds to the relevant outcome analysis. Direct, indirect, and network evidence are distinguished; network certainty is not rated above the most credible contributing evidence. Imprecision is downgraded when the 95% CrI crosses the null and includes clinically important benefit and harm, and sparse/zero-event safety estimates receive additional downgrading when intervals are unstable or extremely wide. TTP is recorded as not quantitatively assessed because the available summaries did not meet the prespecified conversion criteria. The SAE and ILD/pneumonitis GRADE rows correspond to the fixed-effect Bayesian models.

**Location of revision:** Methods - Certainty of Evidence; Results - GRADE Assessment; S2 Appendix, S2-S10 Tables; S3 Appendix GRADE materials.

**Revised text:** “One level was downgraded for reliance on indirect evidence when no direct randomized comparison was available; one level was downgraded for imprecision when the 95% CrI crossed the null and included clinically important benefit and harm; rare-event estimates were downgraded when sparse or zero events produced unstable or extremely wide intervals; and TTP certainty was not quantitatively rated because the available evidence did not support the prespecified quantitative synthesis.”

## Minor Concern 5. Subgroup Analyses

Key subgroup findings should be summarized more prominently, geographic heterogeneity should be discussed, and truncated labels in the supplementary forest plots should be corrected.

**Response:** We retained the expanded PFS and OS subgroup reporting while clarifying that all subgroup analyses are trial-level and exploratory. Numerical estimates are summarized for line of therapy, baseline brain-metastasis prevalence, prior pertuzumab exposure, and geographic region, with emphasis on limited precision. The Discussion highlights that T-DXd was absent from the Asia-dominant networks and that the Asia-dominant T-DM1 versus Lap-Cap OS estimate crossed the null. Figure S1 and Figure S2 labels and legends are complete and are supplied as separate Supporting Information figures.

**Location of revision:** Results - PFS and OS sections; Discussion - clinical interpretation; Figure S1 and Figure S2 and their legends.

**Revised text:** “Geographic subgroup findings warrant caution: T-DXd was absent from the Asia-dominant networks, and the Asia-dominant T-DM1 versus Lap-Cap OS estimate crossed the null. All subgroup analyses were trial-level and exploratory and should not be interpreted as patient-level treatment interactions.”

We again thank the Academic Editor and both reviewers for their thoughtful comments. The revised manuscript and Supporting Information consistently report the same evidence base, outcome definitions, model specifications, and effect estimates across the text, S2 GRADE tables, Figures S1-S9, and S3 analysis files. We respectfully submit the revised manuscript for further consideration.

**Sincerely,**

Gang Hu, on behalf of all authors
