# Supplementary material for: Efficacy and safety of T-DXd versus Other HER2-targeted therapies in second-line and later settings for HER2-positive metastatic breast cancer: A Bayesian network meta-analysis
Source: PLoS One. 2026 Sep 29;21(9):e0358540. doi: 10.1371/journal.pone.0358540 (PMC13623098; doi:10.1371/journal.pone.0358540)
Supplement: Supplementary file 7 [file pone.0358540.s017.docx]

**Manuscript ID: PONE-D-26-23670R1**

Title: Efficacy and Safety of T-DXd Versus Other HER2-Targeted Therapies in Second-Line and Later Settings for HER2-Positive Metastatic Breast Cancer: A Bayesian Network Meta-Analysis

Dear Dr. Shoari,

We sincerely thank you for the careful second-round evaluation of our manuscript. We also thank Reviewer #2 for confirming that all previous comments have been addressed and that the manuscript is technically sound, the statistical analysis is appropriate, the underlying data are available, and the presentation is clear. In response to the Academic Editor’s additional comments, we made focused revisions to improve scope accuracy, make the limitations of the T-DXd survival rankings explicit at the point of the primary efficacy claims, strengthen the reproducibility package, simplify diagnostic-number precision, explain an identical subgroup point estimate, standardize reference formatting, and complete a final cross-check of the figures and Supporting Information. S3 Appendix was expanded to provide the analysis materials, model specifications, code, outputs, and documentation required for independent reproduction. Changes in the marked manuscript are shown using Word Track Changes.

# Academic Editor Comments

## 1. Title and scope consistency

**Editor comment:** Reconcile the short title in the submission system with the short title in the manuscript; revise the full title and/or abstract so that the evidence base is accurately described as including both true second-line and mixed second-/later-line populations; ensure consistent wording across the title page, abstract, and keywords where relevant.

**Response:** We agree. We revised the full title so that it explicitly refers to “Second-Line and Later Settings” rather than implying that the entire evidence base is restricted to second-line treatment. The Abstract already stated that therapies used in “second-line and later settings” were compared, so that wording was retained. The manuscript short title remains “Second-line and later HER2 therapy NMA,” and the same short title should be entered in the submission system. The Keywords did not contain an inaccurate second-line-only restriction and therefore required no change.

**Location of revision:** Title page; Abstract - Background. Revised full title: “Efficacy and Safety of T-DXd Versus Other HER2-Targeted Therapies in Second-Line and Later Settings for HER2-Positive Metastatic Breast Cancer: A Bayesian Network Meta-Analysis.”

## 2. Prominence of the T-DXd subgroup limitation

**Editor comment:** Add an explicit caveat in the Abstract and Results where T-DXd survival/SUCRA statements are presented, noting that T-DXd is not represented in the second-line-only OS network or the Asia-dominant PFS/OS networks, and make clear that the headline survival ranking is driven by mixed-line, non-Asia-dominant trials.

Response: We agree and have made this limitation explicit at the point of the principal efficacy statements rather than leaving it only in the Discussion/Limitations. The Abstract Results now states that T-DXd was not represented in the second-line-only OS network or the Asia-dominant PFS/OS networks and that its survival ranking reflects evidence primarily from mixed-line, non-Asia-dominant trials. The Abstract Conclusion was similarly qualified. In the PFS and OS Results sections, the SUCRA ranking statements are immediately followed by outcome-specific caveats that define the evidence base to which the rankings apply.

**Location of revision:** Abstract - Results and Conclusion; Results - Progression-free survival; Results - Overall survival.

## 3. Completeness and reproducibility of S3 Appendix

**Editor comment:** Confirm that S3 Appendix contains a runnable, self-contained analytic package including extraction data, R and JAGS scripts, analysis inputs and outputs, and sufficient documentation for a third party to reproduce the reported HRs/ORs, including fixed-effect models for SAE, ILD/pneumonitis, and DoR.

**Response:** We substantially strengthened S3 Appendix as a runnable, self-contained analytic package. The package contains the extraction and analysis data, R scripts, standalone JAGS model files, Primary and Secondary analysis inputs and outputs, reference outputs, updated README documentation, a SHA-256 file manifest, and a root-level one-command reproducibility entry point. The package includes the analysis structures used to generate the reported results, including the report-level PFS/OS and confirmed ORR networks and outcome-specific fixed-effect Bayesian models for sparse safety outcomes and DoR. The root script checks the R/JAGS environment and required packages, runs the complete Primary and Secondary workflows, regenerates the analysis outputs and supplementary figures, and compares generated manuscript-result outputs with the included reference values using prespecified numerical tolerances. We also successfully executed the complete package end-to-end in a Windows 11 x64 environment using R 4.6.1 and JAGS 4.3.2; the run log, reproducibility status file, and session information are included in Reproducibility_Check/.

**Location of revision:** S3 Appendix (Supplementary Data and Code), including run_reproducibility.R; Primary and Secondary extraction/analysis data and README files; standalone JAGS model files; reference-output comparison scripts; FILE_MANIFEST.csv; and Reproducibility_Check/ containing the successful run log, reproducibility_status.csv, and sessionInfo.txt.

## 4. Numerical precision

**Editor comment:** Round model diagnostics (for example DIC values and differences) to 1-2 decimal places unless greater precision is analytically necessary.

**Response:** We agree. We simplified model-diagnostic reporting in the main text to two decimal places. For PFS, the consistency and UME DIC values are now reported as 17.50 and 17.49, and the uninformative four-decimal difference was removed. For OS, the values are 14.90 and 14.81 with a difference of 0.08. For confirmed ORR, the values are 34.33 and 34.26. Full numerical precision remains available in machine-readable analysis outputs where appropriate.

**Location of revision:** Results - Progression-free survival; Results - Overall survival; Results - Confirmed objective response rate.

## 5. Clarification of identical Lap-Cap versus PTN-Cap point estimates

**Editor comment:** Explain why the Lap-Cap versus PTN-Cap PFS HR is 2.66 in both the overall and second-line-only analyses even though the CrIs differ.

**Response:** We added the requested explanation immediately after the subgroup estimate. All trials contributing to the PTN-Cap comparison were second-line-only; therefore, the same evidence determines the central Lap-Cap versus PTN-Cap point estimate in the overall and second-line-only analyses. The CrIs differ because the subgroup-specific estimate is obtained within the restricted second-line-only network.

Location of revision: Results - Progression-free survival. Added sentence: “The identical Lap-Cap versus PTN-Cap point estimate in the overall and second-line-only analyses arose because all trials contributing to the PTN-Cap comparison were second-line-only; the different CrIs reflect estimation within the subgroup-specific network.”

## 6. Reference formatting

**Editor comment:** Correct Reference 22 to use “PLOS ONE” and check the entire reference list for consistent journal-name capitalization and PLOS style.

**Response:** We corrected Reference 22 from “PloS one” to “PLOS ONE” and performed a complete journal-name consistency check. We also standardized three visibly inconsistent journal-name entries to the same abbreviated style used throughout the reference list: “Lancet Oncol.” (Reference 5), “Cancer Drug Resist.” (Reference 24), and “J Transl Med.” (Reference 28). The bibliographic content, authors, years, volume/issue information, and DOIs were otherwise unchanged.

**Location of revision:** References 5, 22, 24, and 28.

## 7. Author affiliation check

**Editor comment:** Verify that Yu Xiao’s affiliation with the Sichuan Academy of Social Sciences is correct as typeset; if incorrect, update it, and if correct, confirm it in the response letter.

**Response:** We confirm that Yu Xiao’s affiliation with the Sichuan Academy of Social Sciences, Chengdu, Sichuan 610071, China, is correct as presented in the manuscript. No affiliation change is required.

**Location of revision: Title page. The affiliation is retained as presented in the manuscript.**

## 8. Final figure/table and Supporting Information cross-check

**Editor comment:** Perform a final proofread to ensure that all in-text callouts for Figures 1-5, Figures S1-S9, and appendices match the Supporting Information file list and legends, and correct minor inconsistencies before typesetting.

**Response:** We completed a global cross-check of the manuscript, standalone Figure Legends file, separate figure files, S1 materials, S2 Appendix, and revised S3 Appendix. Figures 1-5 and S1-S9 are all present with unique, continuous numbering and corresponding legends. The S3-S9 figure files were cross-checked against the corresponding files generated in the revised S3 package and were identical. The S2 Appendix table numbering and Supporting Information callouts were also checked. Minor legend wording/capitalization differences between the manuscript and standalone Figure Legends file were harmonized, including capitalization of “Convergence diagnostics” in the S3 Fig legend.

**Location of revision:** Figure Legends file; supporting-information legends in the manuscript; Figures 1-5 and S1-S9; S1-S3 Supporting Information materials.

# Reviewer #2

We sincerely thank Reviewer #2 for the careful re-evaluation of our manuscript and for confirming that all comments from the previous round have been adequately addressed. We particularly appreciate the reviewer’s positive assessment of the technical soundness of the study, the rigor and appropriateness of the statistical analysis, the availability of the underlying data, and the clarity of the manuscript. No additional reviewer-specific revision was requested in this round.

We again thank the Academic Editor and Reviewer #2 for their careful and constructive evaluation. All requested editorial revisions have now been incorporated into the revised manuscript and Supporting Information. We respectfully submit the revised materials for further consideration.

Sincerely,

Gang Hu, on behalf of all authors
