# Supplementary material for: Efficacy and safety of T-DXd versus Other HER2-targeted therapies in second-line and later settings for HER2-positive metastatic breast cancer: A Bayesian network meta-analysis
Source: PLoS One. 2026 Sep 29;21(9):e0358540. doi: 10.1371/journal.pone.0358540 (PMC13623098; doi:10.1371/journal.pone.0358540)
Supplement: Peer review history

# Peer review: Decision Letter 0

**DOI:** 10.1371/journal.pone.0358540.r001

**Authors:** Alireza Shoari

21 Jul 2026

Dear Dr. Hu,

Thank you for submitting your manuscript to PLOS ONE. After careful consideration, we feel that it has merit but does not fully meet PLOS ONE’s publication criteria as it currently stands. Therefore, we invite you to submit a revised version of the manuscript that addresses the points raised during the review process.

Please submit your revised manuscript by Sep 19 2026 11:59PM. If you will need more time than this to complete your revisions, please reply to this message or contact the journal office at plosone@plos.org. When you're ready to submit your revision, log on to https://www.editorialmanager.com/pone/ and select the 'Submissions Needing Revision' folder to locate your manuscript file.

- A letter that responds to each point raised by the academic editor and reviewer(s). You should upload this letter as a separate file labeled 'Response to Reviewers'.
- A marked-up copy of your manuscript that highlights changes made to the original version. You should upload this as a separate file labeled 'Revised Manuscript with Track Changes'.
- An unmarked version of your revised paper without tracked changes. You should upload this as a separate file labeled 'Manuscript'.

If you would like to make changes to your financial disclosure, please include your updated statement in your cover letter. Guidelines for resubmitting your figure files are available below the reviewer comments at the end of this letter.

If applicable, we recommend that you deposit your laboratory protocols in protocols.io to enhance the reproducibility of your results. Protocols.io assigns your protocol its own identifier (DOI) so that it can be cited independently in the future. For instructions see: https://journals.plos.org/plosone/s/submission-guidelines#loc-laboratory-protocols. Additionally, PLOS ONE offers an option for publishing peer-reviewed Lab Protocol articles, which describe protocols hosted on protocols.io. Read more information on sharing protocols at https://plos.org/protocols?utm_medium=editorial-email&utm_source=authorletters&utm_campaign=protocols.

As the corresponding author, your ORCID iD is verified in the submission system and will appear in the published article. PLOS supports the use of ORCID, and we encourage all coauthors to register for an ORCID iD and use it as well. Please encourage your coauthors to verify their ORCID iD within the submission system before final acceptance, as unverified ORCID iDs will not appear in the published article. *Only*  the individual author can complete the verification step; PLOS staff *cannot*  verify ORCID iDs on behalf of authors.

We look forward to receiving your revised manuscript.

Kind regards,

Alireza Shoari

Academic Editor

PLOS One

Journal Requirements:

When submitting your revision, we need you to address these additional requirements.

1.Please ensure that your manuscript meets PLOS ONE's style requirements, including those for file naming. The PLOS ONE style templates can be found at

https://journals.plos.org/plosone/s/file?id=wjVg/PLOSOne_formatting_sample_main_body.pdf and

https://journals.plos.org/plosone/s/file?id=ba62/PLOSOne_formatting_sample_title_authors_affiliations.pdf

2. Please note that funding information should not appear in any section or other areas of your manuscript. We will only publish funding information present in the Funding Statement section of the online submission form. Please remove any funding-related text from the manuscript.

3. Thank you for stating the following financial disclosure:

“This study was supported by Sichuan Provincial People's Hospital, University of Electronic Science and Technology of China, China. Grant no. 2023QN08.”

Please state what role the funders took in the study.  If the funders had no role, please state: "The funders had no role in study design, data collection and analysis, decision to publish, or preparation of the manuscript."

If this statement is not correct you must amend it as needed.

Please include this amended Role of Funder statement in your cover letter; we will change the online submission form on your behalf.

4. In the online submission form, you indicated that “All raw data and code are available upon request.”

All PLOS journals now require all data underlying the findings described in their manuscript to be freely available to other researchers, either 1. In a public repository, 2. Within the manuscript itself, or 3. Uploaded as supplementary information.

This policy applies to all data except where public deposition would breach compliance with the protocol approved by your research ethics board. If your data cannot be made publicly available for ethical or legal reasons (e.g., public availability would compromise patient privacy), please explain your reasons on resubmission and your exemption request will be escalated for approval.

5. We notice that your supplementary figures are included in the manuscript file. Please remove them and upload them with the file type 'Supporting Information'. Please ensure that each Supporting Information file has a legend listed in the manuscript after the references list.

6. If the reviewer comments include a recommendation to cite specific previously published works, please review and evaluate these publications to determine whether they are relevant and should be cited. There is no requirement to cite these works unless the editor has indicated otherwise.

[Note: HTML markup is below. Please do not edit.]

Reviewers' comments:

Reviewer's Responses to Questions

**Comments to the Author**

1. Is the manuscript technically sound, and do the data support the conclusions?

Reviewer #1: Yes

Reviewer #2: Yes

**********

2. Has the statistical analysis been performed appropriately and rigorously?

Reviewer #1: Yes

Reviewer #2: Yes

**********

3. Have the authors made all data underlying the findings in their manuscript fully available?

The PLOS Data policy

Reviewer #1: Yes

Reviewer #2: Yes

**********

4. Is the manuscript presented in an intelligible fashion and written in standard English?

Reviewer #1: Yes

Reviewer #2: Yes

**********

Reviewer #1: This manuscript presents a well-structured and methodologically sound study addressing an important and timely research question. The authors demonstrate strong analytical rigor, and the findings are clearly presented and supported by appropriate evidence. The discussion is balanced, with thoughtful interpretation of results in the context of existing literature. Overall, the work makes a valuable contribution to the field and is suitable for publication with minor revisions for clarity and formatting.

Reviewer #2: OVERALL RECOMMENDATION

This manuscript presents a timely and clinically important Bayesian network meta-analysis (NMA) comparing trastuzumab deruxtecan (T-DXd) with other HER2-targeted therapies in second-line HER2-positive metastatic breast cancer. The study addresses a significant evidence gap by incorporating T-DXd—the current standard of care—into a comprehensive comparative framework. While the topic is highly relevant and the analytical approach is generally sound, several methodological, reporting, and interpretive issues require substantial revision before the manuscript can be considered for publication in PLOS ONE. The current version has notable deficiencies in data transparency, statistical reporting consistency, and conclusion-support alignment that must be addressed.

MAJOR CONCERNS (Must be addressed for publication)

1. DATA AVAILABILITY STATEMENT — NON-COMPLIANCE WITH PLOS ONE POLICY

• The Data Availability Statement on page 6 states: 'All raw data and code are available upon request.' This is unacceptable under PLOS ONE's data policy, which requires that "all data underlying the findings be made fully available and without restriction."

• Specific issues:

• • PLOS ONE mandates public deposition of data in recognized repositories (e.g., Zenodo, Figshare, Dryad, GitHub, or Open Science Framework) with DOIs or accession numbers provided.

• • "Available upon request" does not meet the journal's requirement for unrestricted public access.

• The R code used for Bayesian NMA (gemtc package, MCMC simulations, network plots) must be deposited in a public repository with a persistent identifier.

• • The extracted data from the 16 trials should be provided as a supplementary dataset or deposited in a repository.

• REQUIRED ACTION: Revise the Data Availability Statement to specify the public repository name and DOI/accession number and ensure all data and code are uploaded before resubmission.

2. INCONSISTENCY BETWEEN ABSTRACT AND MAIN TEXT RESULTS

• The Abstract (page 1, lines 59-67) states: "Trastuzumab deruxtecan provided the greatest survival benefit, improving progression-free survival (hazard ratio 0.30, 95% credible interval 0.19–0.49) and overall survival (hazard ratio 0.66, 95% credible interval 0.50–0.84) versus other regimens, with fewer serious adverse events (odds ratio 0.65, 95% credible interval 0.46–0.96)."

• However, in the Results section:

• • Page 22, lines 279-284 (PFS): T-DXd vs. T-DM1 HR = 0.30 (0.19–0.49) — this is correct.

• • Page 22, lines 289-293 (OS): T-DXd vs. T-DM1 HR = 0.66 (0.50–0.84) — this is correct.

• • Page 23, lines 300-305 (SAEs): The abstract claims T-DXd had "fewer serious adverse events" with OR 0.65 (0.46–0.96). However, the Results section states: "Direct evidence suggested that T-DM1 reduced the odds of SAEs versus Lap-Cap (OR 0.65, 95% CrI 0.48–0.89; p = 0.0069). NMA was consistent with the direct comparison, indicating that T-DM1 was favored versus Lap-Cap (OR 0.65, 95% CrI 0.46–0.96) and PTN-Cap (OR 0.41, 95% CrI 0.19–0.92), and that T-DXd was favored versus PTN-Cap (OR 0.39, 95% CrI 0.16–0.97)."

• CRITICAL ISSUE: The OR of 0.65 (0.46–0.96) cited in the abstract for T-DXd and SAEs actually refers to T-DM1 vs. Lap-Cap, NOT T-DXd. The abstract misattributes this statistic. T-DXd vs. PTN-Cap for SAEs was OR 0.39 (0.16–0.97). The abstract's claim that T-DXd had "fewer serious adverse events" with OR 0.65 is INCORRECT and misleading.

• REQUIRED ACTION: Correct the abstract to accurately reflect the SAE findings. The abstract should either cite the correct T-DXd-specific SAE comparison or rephrase the safety claim to accurately represent the network findings.

3. MISSING PRISMA-NMA CHECKLIST AND FLOW DIAGRAM ISSUES

• The manuscript states adherence to PRISMA-NMA guidelines (page 14, line 141) and includes a PRISMA flow diagram (Figure 1, page 41). However:

• The PRISMA-NMA checklist is not provided as supporting information, which is mandatory for systematic reviews and meta-analyses submitted to PLOS ONE.

• Figure 1 (PRISMA flow diagram) shows "Records excluded (n=761)" with subcategories totaling 761, but the full-text exclusion reasons (n=723) only list four categories totaling 723. However, 14 + 533 + 6 + 170 = 723, which mathematically checks out. The issue is that the diagram shows 739 full-text articles assessed, with 723 excluded, leaving 16 included — but the reasons for the 723 exclusions are incomplete. The category "Wrong intervention (n=533)" is extremely large and warrants breakdown.

• • The flow diagram should also indicate the number of studies excluded for each specific reason to enhance transparency.

• REQUIRED ACTION:

• (a) Include the completed PRISMA-NMA checklist as supporting information.

• (b) Provide a more detailed breakdown of the 533 studies excluded for "wrong intervention."

• (c) Ensure the flow diagram aligns with PRISMA 2020 updated guidance for NMAs.

4. SEARCH DATE DISCREPANCY AND INCOMPLETE SEARCH STRATEGY

• The Methods section (page 15, lines 148-150) states: "A systematic literature search was conducted... from their inception to October 11, 2025."

• Issues:

• • The search was conducted until October 11, 2025, but the manuscript was submitted in 2026 (manuscript number PONE-D-26-23670 suggests 2026 submission). There is a significant gap between the search date and submission. Have the authors conducted an updated search to capture trials published between October 2025 and the submission date? If not, this should be acknowledged as a limitation.

• • The complete search syntax is referenced as being "provided in the supplementary materials" (page 15, line 152), but the supplementary materials are not accessible in this draft. PLOS ONE requires the full search strategy to be included in the main text or as supporting information.

• • No language restrictions were applied — this is good, but the authors should confirm whether non-English literature was actually identified and how it was handled.

• REQUIRED ACTION:

• (a) Clarify whether an updated search was performed and report any additional trials identified.

• (b) Include the complete search strategy for all databases in the main text or as supporting information.

• (c) If no updated search was conducted, explicitly state this as a limitation.

MINOR CONCERNS (Should be addressed to improve manuscript quality)

1. Abstract Length and Structure

• The abstract is 3975 words (page 9, line 2), which far exceeds PLOS ONE's 300-word limit for abstracts. The current abstract is essentially a mini-paper. It must be condensed to ≤300 words, following the standard structure: Background, Methods, Results, Conclusion. The detailed statistics should be moved to the main text or presented more concisely.

2. Figure and Table Legends

• Figure 4 and Figure 5 legends are identical (both state "Network meta-analysis results for [PFS/OS]" with identical subpanel descriptions). The legends should be specific to each figure's content.

• Supplementary Figures 3-9 have truncated or garbled legends (e.g., "# upplementary Figure 3. Network meta-analysis results for Su"). These need correction.

• • Figure 2 (risk of bias summary) and Figure 3 (individual risk of bias) are clear, but the color coding should be explicitly defined in the legend (green = low, yellow = unclear, red = high).

3. Statistical Reporting Consistency

• • The manuscript uses both "95% credible interval" and "95% CrI" interchangeably. Standardize to one format throughout.

• • Some p-values are reported (e.g., p = 0.0069 for SAEs), but Bayesian NMAs typically do not use p-values. Clarify whether these are from direct pairwise meta-analyses (frequentist) or from the Bayesian framework. If frequentist p-values are reported alongside Bayesian CrIs, explain the rationale.

• • The I2 statistic is reported for heterogeneity, but I2 is a frequentist measure. In Bayesian NMA, between-study variance (tau²) or predictive intervals are more appropriate. Clarify the statistical framework used for heterogeneity assessment.

4. GRADE Assessment Details

• Page 26, lines 362-380, describes GRADE assessments, but:

• • The GRADE ratings are referenced as "Supplementary Table 2" through "Supplementary Table 10," but these tables are not accessible in the current draft.

• The text states, "PFS was rated High for the direct estimate and Moderate for the network estimate"—this is unusual because network estimates are typically rated lower than direct estimates due to indirectness. The rationale for this specific rating should be explained.

• • The justification for downgrading due to "imprecision" should be more specific (e.g., what width of CrI triggered the downgrade?).

5. Subgroup Analyses

• Page 19, lines 236-239, mentions prespecified subgroup analyses for PFS and OS by baseline brain metastases, line of therapy, geographic region, and prior pertuzumab exposure. The results are referenced as "Supplementary Figure 1" and "Supplementary Figure 2" (pages 47-48), but:

• • The subgroup analyses are only briefly mentioned in the main text (page 22, lines 293-298 for OS; page 22, lines 284-288 for PFS). Key findings should be summarized more prominently.

• • Some subgroup results are clinically important (e.g., Asia-dominant comparisons between T-DM1 and Lap-Cap were uncertain: HR 1.08, 95% CrI 0.58–2.02). This geographic heterogeneity deserves discussion.

• • The forest plots in Supplementary Figures 1 and 2 have truncated labels (e.g., "Forest plot of PFS stratified by trials with a higher prevalence of baseline brain metastases"). Correct the truncation.

**********

what does this mean?). If published, this will include your full peer review and any attached files.

If you choose “no”, your identity will remain anonymous but your review may still be made public.

**Do you want your identity to be public for this peer review?**  For information about this choice, including consent withdrawal, please see our Privacy Policy

Reviewer #1: No

Reviewer #2: **Yes:**  DR. MURSHIDUL AREFIN

**********

[NOTE: If reviewer comments were submitted as an attachment file, they will be attached to this email and accessible via the submission site. Please log into your account, locate the manuscript record, and check for the action link "View Attachments". If this link does not appear, there are no attachment files.]

To ensure your figures meet our technical requirements, please review our figure guidelines: https://journals.plos.org/plosone/s/figures

You may also use PLOS’s free figure tool, NAAS, to help you prepare publication quality figures: https://journals.plos.org/plosone/s/figures#loc-tools-for-figure-preparation.

NAAS will assess whether your figures meet our technical requirements by comparing each figure against our figure specifications.

# Author comment: Author response to Decision Letter 1

**DOI:** 10.1371/journal.pone.0358540.r002

13 Aug 2026

Response to the Academic Editor and Reviewers

Manuscript ID PONE-D-26-23670

Title Efficacy and Safety of T-DXd Versus Other HER2-Targeted Therapies in Second-Line HER2-Positive Metastatic Breast Cancer: A Bayesian Network Meta-Analysis

Dear Dr. Shoari,

We sincerely thank you and the reviewers for the careful and constructive evaluation of our manuscript. We revised the manuscript to strengthen methodological reporting, statistical consistency, and coherence across the main text, Supporting Information, figures, and analysis code. The literature search remains updated through July 24, 2026, and the evidence base comprises 16 reports representing seven independent randomized trial families and 3,339 unique randomized participants. Secondary outcomes were analyzed using consistent endpoint definitions and event coding. Sparse SAE and ILD/pneumonitis networks were evaluated with fixed-effect Bayesian models, DoR was analyzed exploratorily using the prespecified summary-statistic conversion, and TTP was reported descriptively because the available data did not satisfy the criteria for quantitative synthesis. The PRISMA materials, GRADE profiles, figure legends, and S3 data/code package were revised accordingly. All changes are reflected in the marked manuscript. Our point-by-point responses are provided below.

Academic Editor and Journal Requirements

Journal Requirement 1. Please ensure that the manuscript meets PLOS ONE style requirements, including file naming.

Response: We revised the manuscript structure and formatting to conform more closely to PLOS ONE requirements. We prepared clean and marked manuscript files, removed supplementary figure images from the manuscript body, organized Figure 1-5 and Figure S1-S9 as separate figure files, and standardized the Supporting Information file names for the revised submission package.

Location of revision: Manuscript structure; Figure Legend file; separately prepared Figure 1-5 and Figure S1-S9 files; S1-S3 Supporting Information materials.

Journal Requirement 2. Funding information should not appear in the manuscript and should be reported only in the online Funding Statement.

Response: We removed funding-related disclosure text from the manuscript. The manuscript retains only the CRediT author-contribution role “funding acquisition,” which describes an author contribution and is not a funding disclosure. The funding source will be reported only in the online submission form, as requested.

Location of revision: Declarations and Author Contributions sections.

Journal Requirement 3. Please clarify the role of the funders and include the amended Role of Funder statement in the cover letter.

Response: We confirm that the funders had no role in the conduct or reporting of the study. The following statement will be included in the cover letter and the online submission record: “The funders had no role in study design, data collection and analysis, decision to publish, or preparation of the manuscript.”

Journal Requirement 4. “All raw data and code are available upon request” does not comply with the PLOS data policy. Data underlying the findings must be publicly available through a repository, the manuscript, or Supporting Information.

Response: We agree and have replaced the “available upon request” statement. The revised Data Availability Statement now states that the data and code used in this study are included in S3 Appendix (Supplementary Data and Code).

Location of revision: Data Availability declaration; Supporting Information file list; S3 Appendix. Supplementary Data and Code.

Revised text: “The data and code used in this study are included in S3 Appendix (Supplementary Data and Code).”

Journal Requirement 5. Supplementary figures should be removed from the manuscript file, uploaded as Supporting Information, and accompanied by legends after the References.

Response: We removed all supplementary figure images from the manuscript body and prepared Figure S1-S9 as separate Supporting Information figure files. Complete legends for Figure 1-5 and S1-S9 Figs are provided in the updated Figure Legend file and the S1-S9 Fig legends are also retained after the References in the manuscript.

Location of revision: Figure Legend file; Supporting information legends after the References; separate Figure S1-S9 files.

Journal Requirement 6. Reviewer-recommended citations should be evaluated for relevance; there is no requirement to cite them unless directed by the editor.

Response: We reviewed the reference list for relevance and consistency. The reviewers did not require citation of specific publications. We added or retained references only where they directly supported the revised methodological and clinical interpretation.

Reviewer 1

The manuscript is well structured and methodologically sound and is suitable for publication with minor revisions for clarity and formatting.

Response: We thank the reviewer for this positive assessment. In response to the request for greater clarity and formatting consistency, we refined the title and abstract, distinguished publication-level reports from independent randomized trial families, clarified outcome-specific network connectivity, standardized statistical terminology and secondary-outcome definitions, expanded the methods and limitations, updated the figure legends, and reorganized the Supporting Information. We believe these changes substantially improve clarity, reproducibility, and presentation.

Reviewer 2

General response: We appreciate the reviewer's detailed assessment. In response to these comments, we repeated the secondary-outcome analyses using uniform endpoint definitions and event coding. This yielded revised safety estimates and showed that the available TTP summaries did not satisfy the prespecified criteria for quantitative synthesis. We therefore revised the manuscript, S2 GRADE tables, figures, and S3 analysis package so that all materials report consistent outcome definitions, model specifications, effect estimates, and interpretations.

Major Concern 1. Data Availability Statement

The statement “All raw data and code are available upon request” is not compliant. Data, extracted trial information, and R code should be made publicly available with unrestricted access.

Response: We agree that the original statement was inadequate and have removed it. The revised submission includes the extracted data, analysis inputs, reproducible R/JAGS scripts, manifests, and analysis outputs in S3 Appendix (Supplementary Data and Code), and the Data Availability Statement has been revised accordingly.

Location of revision: Data Availability declaration; Supporting Information file list; S3 Appendix. Supplementary Data and Code.

Revised text: “The data and code used in this study are included in S3 Appendix (Supplementary Data and Code).”

Major Concern 2. Inconsistency Between the Abstract and Main-Text Safety Results

The abstract incorrectly attributed OR 0.65 (95% CrI 0.46-0.96) to T-DXd for serious adverse events; this estimate actually represents T-DM1 versus Lap-Cap. The safety statement was misleading and should be corrected.

Response: We agree and thank the reviewer for identifying the original safety attribution error. Following this observation, the secondary safety outcomes were analyzed with toxicity occurrence consistently coded as the event, and the sparse safety networks were re-estimated using the prespecified Bayesian framework. The previously reported SAE estimates of 0.65 and 0.95 were therefore replaced. In the fixed-effect Bayesian SAE network, neither T-DM1 versus Lap-Cap nor T-DXd versus T-DM1 showed a clear difference. The fixed-effect Bayesian ILD/pneumonitis analysis continued to show substantially higher odds with T-DXd than with T-DM1. The Abstract, Results, Discussion, Conclusion, GRADE profiles, Figures S7-S8, and S3 code/output files consistently report these estimates and interpretations.

Location of revision: Abstract - Results and Conclusion; Results - any-grade AE, SAE, ILD/pneumonitis, and LVD sections; Discussion; Conclusion; S2 Appendix; Figures S6-S9; S3 Appendix.

Revised text: “Any-grade AE estimates did not indicate a safety advantage for T-DXd. SAE comparisons were also inconclusive: T-DM1 vs Lap-Cap OR 0.90 (95% CrI 0.67-1.21) and T-DXd vs T-DM1 OR 1.30 (0.87-1.93). T-DXd had higher ILD/pneumonitis odds than T-DM1 (OR 5.55, 2.79-12.16); LVD differences were unclear.”

Major Concern 3. PRISMA-NMA Checklist and Flow Diagram

A completed PRISMA-NMA checklist was missing. The flow diagram required a more transparent breakdown of exclusions, particularly the large “wrong intervention” category, and alignment with PRISMA 2020 guidance.

Response: We addressed all three points. First, the completed PRISMA 2020 checklist and PRISMA-NMA checklist are now supplied as separate S1 Supporting Information files, and the complete search strategies are supplied as S1 Appendix Search Strategies. Second, after updating the search, we rebuilt the PRISMA 2020 flow diagram using the updated search counts rather than the previous broad “wrong intervention” category. The revised flow reports database-specific yields, duplicate removal, title/abstract exclusions with explicit categories, and full-text exclusions with explicit reasons. Third, the Study Selection text was rewritten to match Figure 1 exactly and distinguishes 16 publication-level reports from seven independent randomized trial families.

Location of revision: Methods - Reporting framework and Literature Search; Results - Study Selection; Figure 1; S1 PRISMA-NMA checklist-2020 checklist; S1 PRISMA-NMA checklist-NMA checklist; S1 Appendix Search Strategies.

Revised text: “The updated searches identified 3,591 records... After removal of 742 duplicates, 2,849 records were screened... Thirty-two full-text articles were assessed. Sixteen were excluded for five explicit reasons. Sixteen reports representing seven independent randomized trial families and 3,339 unique randomized participants were included.”

Major Concern 4. Search-Date Discrepancy and Search Strategy

Please clarify whether the search was updated, provide the complete search strategies for all databases, and explain how non-English literature was handled. If no update was performed, the gap should be acknowledged as a limitation.

Response: We performed an updated search through July 24, 2026, before resubmission. The update retrieved 3,591 records in total and did not identify an additional eligible randomized trial family. Complete reproducible strategies for PubMed, Embase, Web of Science Core Collection, and CENTRAL are provided in S1 Appendix Search Strategies. No language restrictions were applied, and all 16 included full-text reports were published in English. Conference abstracts and proceedings were captured by the broad searches but were not eligible for inclusion as full-text article reports under the prespecified criteria.

Location of revision: Abstract - Methods; Methods - Literature Search; Results - Study Selection; Discussion; Figure 1; S1 Appendix Search Strategies.

Revised text: “An updated search was completed on July 24, 2026... No language restrictions were applied, and all 16 included reports were published in English... The evidence base comprised 16 reports representing seven independent randomized trial families.”

Minor Concern 1. Abstract Length and Structure

The abstract substantially exceeded the PLOS ONE 300-word limit and should be condensed into Background, Methods, Results, and Conclusion.

Response: We rewrote the abstract as a structured 300-word abstract excluding the four section headings, meeting the PLOS ONE limit. The revised version reports the report/trial-family structure, primary comparative estimates, secondary efficacy findings, safety results, and a cautious conclusion.

Location of revision: Abstract.

Minor Concern 2. Figure and Table Legends

The legends for Figures 4 and 5 were insufficiently differentiated; supplementary figure legends were truncated or garbled; and the risk-of-bias color coding should be defined explicitly.

Response: We updated the standalone Figure Legend file to cover Figure 1-5 and Figure S1-S9 and to correspond with the revised figure set. Figure 4 is explicitly the PFS NMA and Figure 5 the OS NMA. The S3-S9 legends now describe the outcome-specific networks and models directly: exploratory Bayesian analysis for DoR, descriptive reporting of TTP, event-based interpretation of any-grade AE and LVD outcomes, and fixed-effect Bayesian analyses for SAE and ILD/pneumonitis. Figures 2 and 3 define green as low risk, yellow as unclear risk, and red as high risk.

Location of revision: Updated Figure Legend file; Supporting information legends after the References; separate Figure 1-5 and Figure S1-S9 files.

Revised text: “Green indicates low risk, yellow indicates unclear risk, and red indicates high risk.”

Minor Concern 3. Statistical Reporting Consistency

Please standardize “95% credible interval/95% CrI,” distinguish frequentist P values from Bayesian inference, and clarify the use of I² in a Bayesian NMA.

Response: We standardized “95% CrI” after first definition and separated Bayesian inference from frequentist diagnostics. Bayesian NMA conclusions are based on posterior effect estimates and 95% CrIs. Frequentist Q/I² statistics are used only descriptively for direct evidence when meaningfully estimable. We no longer make a blanket claim of low network heterogeneity. Node splitting is performed only when a closed loop provides both direct and indirect evidence; the ORR and DoR networks are tree-shaped and do not permit node splitting. All quantitative outcome networks contain fewer than 10 independent studies, so comparison-adjusted Egger tests are not treated as valid inferential assessments and no P-value-based publication-bias claim is retained. Sparse SAE and ILD/pneumonitis networks use fixed-effect Bayesian models because heterogeneity is not reliably estimable.

Location of revision: Methods - Statistical Analysis, network geometry and convergence; Results - outcome-specific analyses and Publication bias and sensitivity analysis; Discussion - heterogeneity/inconsistency paragraph; S3 Appendix.

Revised text: “Bayesian estimates were reported with 95% CrIs. Random-effects Bayesian models were used where between-study heterogeneity could be estimated with reasonable support. The SAE and ILD/pneumonitis networks used fixed-effect Bayesian models because their sparse structures did not support reliable heterogeneity estimation. Comparison-adjusted Egger tests were not treated as inferential assessments when fewer than 10 independent studies contributed.”

Minor Concern 4. GRADE Assessment Details

The GRADE tables were not accessible, the relationship between direct and network ratings required explanation, and the imprecision criterion should be specified.

Response: We provide complete comparison-level GRADE profiles in S2 Appendix and ensure that each rating corresponds to the relevant outcome analysis. Direct, indirect, and network evidence are distinguished; network certainty is not rated above the most credible contributing evidence. Imprecision is downgraded when the 95% CrI crosses the null and includes clinically important benefit and harm, and sparse/zero-event safety estimates receive additional downgrading when intervals are unstable or extremely wide. TTP is recorded as not quantitatively assessed because the available summaries did not meet the prespecified conversion criteria. The SAE and ILD/pneumonitis GRADE rows correspond to the fixed-effect Bayesian models.

Location of revision: Methods - Certainty of Evidence; Results - GRADE Assessment; S2 Appendix, S2-S10 Tables; S3 Appendix GRADE materials.

Revised text: “One level was downgraded for reliance on indirect evidence when no direct randomized comparison was available; one level was downgraded for imprecision when the 95% CrI crossed the null and included clinically important benefit and harm; rare-event estimates were downgraded when sparse or zero events produced unstable or extremely wide intervals; and TTP certainty was not quantitatively rated because the available evidence did not support the prespecified quantitative synthesis.”

Minor Concern 5. Subgroup Analyses

Key subgroup findings should be summarized more prominently, geographic heterogeneity should be discussed, and truncated labels in the supplementary forest plots should be corrected.

Response: We retained the expanded PFS and OS subgroup reporting while clarifying that all subgroup analyses are trial-level and exploratory. Numerical estimates are summarized for line of therapy, baseline brain-metastasis prevalence, prior pertuzumab exposure, and geographic region, with emphasis on limited precision. The Discussion highlights that T-DXd was absent from the Asia-dominant networks and that the Asia-dominant T-DM1 versus Lap-Cap OS estimate crossed the null. Figure S1 and Figure S2 labels and legends are complete and are supplied as separate Supporting Information figures.

Location of revision: Results - PFS and OS sections; Discussion - clinical interpretation; Figure S1 and Figure S2 and their legends.

Revised text: “Geographic subgroup findings warrant caution: T-DXd was absent from the Asia-dominant networks, and the Asia-dominant T-DM1 versus Lap-Cap OS estimate crossed the null. All subgroup analyses were trial-level and exploratory and should not be interpreted as patient-level treatment interactions.”

We again thank the Academic Editor and both reviewers for their thoughtful comments. The revised manuscript and Supporting Information consistently report the same evidence base, outcome definitions, model specifications, and effect estimates across the text, S2 GRADE tables, Figures S1-S9, and S3 analysis files. We respectfully submit the revised manuscript for further consideration.

Sincerely,

Gang Hu, on behalf of all authors

Attachment Submitted filename: Response to Reviewers.docx

# Peer review: Decision Letter 1

**DOI:** 10.1371/journal.pone.0358540.r003

**Authors:** Alireza Shoari

28 Aug 2026

Dear Dr. Hu,

plosone@plos.org. When you're ready to submit your revision, log on to https://www.editorialmanager.com/pone/ and select the 'Submissions Needing Revision' folder to locate your manuscript file.

- A letter that responds to each point raised by the academic editor and reviewer(s). You should upload this letter as a separate file labeled 'Response to Reviewers'.
- A marked-up copy of your manuscript that highlights changes made to the original version. You should upload this as a separate file labeled 'Revised Manuscript with Track Changes'.
- An unmarked version of your revised paper without tracked changes. You should upload this as a separate file labeled 'Manuscript'.

If you would like to make changes to your financial disclosure, please include your updated statement in your cover letter. Guidelines for resubmitting your figure files are available below the reviewer comments at the end of this letter.

If applicable, we recommend that you deposit your laboratory protocols in protocols.io to enhance the reproducibility of your results. Protocols.io assigns your protocol its own identifier (DOI) so that it can be cited independently in the future. For instructions see: https://journals.plos.org/plosone/s/submission-guidelines#loc-laboratory-protocols. Additionally, PLOS One offers an option for publishing peer-reviewed Lab Protocol articles, which describe protocols hosted on protocols.io. Read more information on sharing protocols at https://plos.org/protocols?utm_medium=editorial-email&utm_source=authorletters&utm_campaign=protocols.

As the corresponding author, your ORCID iD is verified in the submission system and will appear in the published article. PLOS supports the use of ORCID, and we encourage all coauthors to register for an ORCID iD and use it as well. Please encourage your coauthors to verify their ORCID iD within the submission system before final acceptance, as unverified ORCID iDs will not appear in the published article. *Only*  the individual author can complete the verification step; PLOS staff *cannot*  verify ORCID iDs on behalf of authors.

We look forward to receiving your revised manuscript.

Kind regards,

Alireza Shoari

Academic Editor

PLOS One

Journal Requirements:

If the reviewer comments include a recommendation to cite specific previously published works, please review and evaluate these publications to determine whether they are relevant and should be cited. There is no requirement to cite these works unless the editor has indicated otherwise.

**Additional Editor Comments:**

1. Title and scope consistency
  Reconcile the short title in the submission system with the short title in the manuscript file.
2. Revise the full title and/or abstract to accurately reflect that the evidence base includes both true second-line and mixed second-/later-line populations (e.g., “second-line and later HER2 therapy”).
3. Ensure this wording is consistent across the title page, abstract, and keywords if relevant.
4. Prominence of T-DXd subgroup limitation
  In the Abstract and Results sections, add an explicit caveat attached to the T-DXd survival/SUCRA statements noting that:
  T-DXd is not represented in the second-line-only OS network; and
5. T-DXd is not represented in the Asia-dominant PFS/OS networks.
6. Make clear that the headline survival ranking for T-DXd is driven by mixed-line, non–Asia-dominant trials. This caveat should appear where the primary efficacy claims and rankings are stated, not only in the Discussion/Limitations.
7. Completeness and reproducibility of S3 Appendix
  Confirm in your response letter that S3 Appendix contains a runnable, self-contained analytic package, including:
  extraction data;
8. R and JAGS scripts;
9. analysis inputs and outputs;
10. sufficient documentation for a third party to reproduce the reported HRs/ORs (including fixed-effect models for SAE, ILD/pneumonitis, and DoR) without contacting the authors.
11. If any files were incomplete, please update S3 accordingly and clearly state what was corrected.
12. Numerical precision
  Round model diagnostics (e.g., DIC values, differences) to 1–2 decimal places unless additional precision is analytically necessary.
13. For example, DIC = 17.4980 vs 17.4931 (difference 0.0049) should be simplified for readability.
14. Clarify identical point estimates
  Add a brief explanatory sentence regarding the identical Lap-Cap vs PTN-Cap PFS HR (2.66) in the overall and second-line-only analyses (e.g., noting that all PTN-Cap–contributing trials were second-line-only, so the point estimate is shared, while CrIs differ due to subgroup-specific estimation).
15. Reference formatting
  Correct reference 22 to use “PLOS ONE” (not “PloS one”).
16. Check the entire reference list for consistent journal-name capitalization and PLOS style.
17. Author affiliation check
  Verify that Yu Xiao’s affiliation with the Sichuan Academy of Social Sciences is correct as typeset.
18. If incorrect, update the affiliation; if correct, confirm this in your response letter.
19. Final figure/table cross-check
  Perform a final proofread to ensure that all in-text figure/table callouts (Figures 1–5, S1–S9, appendices) match the Supporting Information file list and legends.
20. Correct any minor inconsistencies before typesetting.

[Note: HTML markup is below. Please do not edit.]

Reviewers' comments:

Reviewer's Responses to Questions

**Comments to the Author**

Reviewer #2: All comments have been addressed

**********

2. Is the manuscript technically sound, and do the data support the conclusions?

Reviewer #2: Yes

**********

3. Has the statistical analysis been performed appropriately and rigorously?

Reviewer #2: Yes

**********

4. Have the authors made all data underlying the findings in their manuscript fully available?

The PLOS Data policy

Reviewer #2: Yes

**********

5. Is the manuscript presented in an intelligible fashion and written in standard English?

Reviewer #2: Yes

**********

Reviewer #2: (No Response)

**********

what does this mean?). If published, this will include your full peer review and any attached files.

If you choose “no”, your identity will remain anonymous but your review may still be made public.

**Do you want your identity to be public for this peer review?**  For information about this choice, including consent withdrawal, please see our Privacy Policy

Reviewer #2: **Yes:**  Dr. Murshidul Arefin

**********

[NOTE: If reviewer comments were submitted as an attachment file, they will be attached to this email and accessible via the submission site. Please log into your account, locate the manuscript record, and check for the action link "View Attachments". If this link does not appear, there are no attachment files.]

To ensure your figures meet our technical requirements, please review our figure guidelines: https://journals.plos.org/plosone/s/figures

You may also use PLOS’s free figure tool, NAAS, to help you prepare publication quality figures: https://journals.plos.org/plosone/s/figures#loc-tools-for-figure-preparation.

NAAS will assess whether your figures meet our technical requirements by comparing each figure against our figure specifications.

# Author comment: Author response to Decision Letter 2

**DOI:** 10.1371/journal.pone.0358540.r004

1 Sep 2026

Manuscript ID: PONE-D-26-23670R1

Title: Efficacy and Safety of T-DXd Versus Other HER2-Targeted Therapies in Second-Line and Later Settings for HER2-Positive Metastatic Breast Cancer: A Bayesian Network Meta-Analysis

Dear Dr. Shoari,

We sincerely thank you for the careful second-round evaluation of our manuscript. We also thank Reviewer #2 for confirming that all previous comments have been addressed and that the manuscript is technically sound, the statistical analysis is appropriate, the underlying data are available, and the presentation is clear. In response to the Academic Editor’s additional comments, we made focused revisions to improve scope accuracy, make the limitations of the T-DXd survival rankings explicit at the point of the primary efficacy claims, strengthen the reproducibility package, simplify diagnostic-number precision, explain an identical subgroup point estimate, standardize reference formatting, and complete a final cross-check of the figures and Supporting Information. S3 Appendix was expanded to provide the analysis materials, model specifications, code, outputs, and documentation required for independent reproduction. Changes in the marked manuscript are shown using Word Track Changes.

Academic Editor Comments

1. Title and scope consistency

Editor comment: Reconcile the short title in the submission system with the short title in the manuscript; revise the full title and/or abstract so that the evidence base is accurately described as including both true second-line and mixed second-/later-line populations; ensure consistent wording across the title page, abstract, and keywords where relevant.

Response: We agree. We revised the full title so that it explicitly refers to “Second-Line and Later Settings” rather than implying that the entire evidence base is restricted to second-line treatment. The Abstract already stated that therapies used in “second-line and later settings” were compared, so that wording was retained. The manuscript short title remains “Second-line and later HER2 therapy NMA,” and the same short title should be entered in the submission system. The Keywords did not contain an inaccurate second-line-only restriction and therefore required no change.

Location of revision: Title page; Abstract - Background. Revised full title: “Efficacy and Safety of T-DXd Versus Other HER2-Targeted Therapies in Second-Line and Later Settings for HER2-Positive Metastatic Breast Cancer: A Bayesian Network Meta-Analysis.”

2. Prominence of the T-DXd subgroup limitation

Editor comment: Add an explicit caveat in the Abstract and Results where T-DXd survival/SUCRA statements are presented, noting that T-DXd is not represented in the second-line-only OS network or the Asia-dominant PFS/OS networks, and make clear that the headline survival ranking is driven by mixed-line, non-Asia-dominant trials.

Response: We agree and have made this limitation explicit at the point of the principal efficacy statements rather than leaving it only in the Discussion/Limitations. The Abstract Results now states that T-DXd was not represented in the second-line-only OS network or the Asia-dominant PFS/OS networks and that its survival ranking reflects evidence primarily from mixed-line, non-Asia-dominant trials. The Abstract Conclusion was similarly qualified. In the PFS and OS Results sections, the SUCRA ranking statements are immediately followed by outcome-specific caveats that define the evidence base to which the rankings apply.

Location of revision: Abstract - Results and Conclusion; Results - Progression-free survival; Results - Overall survival.

3. Completeness and reproducibility of S3 Appendix

Editor comment: Confirm that S3 Appendix contains a runnable, self-contained analytic package including extraction data, R and JAGS scripts, analysis inputs and outputs, and sufficient documentation for a third party to reproduce the reported HRs/ORs, including fixed-effect models for SAE, ILD/pneumonitis, and DoR.

Response: We substantially strengthened S3 Appendix as a runnable, self-contained analytic package. The package contains the extraction and analysis data, R scripts, standalone JAGS model files, Primary and Secondary analysis inputs and outputs, reference outputs, updated README documentation, a SHA-256 file manifest, and a root-level one-command reproducibility entry point. The package includes the analysis structures used to generate the reported results, including the report-level PFS/OS and confirmed ORR networks and outcome-specific fixed-effect Bayesian models for sparse safety outcomes and DoR. The root script checks the R/JAGS environment and required packages, runs the complete Primary and Secondary workflows, regenerates the analysis outputs and supplementary figures, and compares generated manuscript-result outputs with the included reference values using prespecified numerical tolerances. We also successfully executed the complete package end-to-end in a Windows 11 x64 environment using R 4.6.1 and JAGS 4.3.2; the run log, reproducibility status file, and session information are included in Reproducibility_Check/.

Location of revision: S3 Appendix (Supplementary Data and Code), including run_reproducibility.R; Primary and Secondary extraction/analysis data and README files; standalone JAGS model files; reference-output comparison scripts; FILE_MANIFEST.csv; and Reproducibility_Check/ containing the successful run log, reproducibility_status.csv, and sessionInfo.txt.

4. Numerical precision

Editor comment: Round model diagnostics (for example DIC values and differences) to 1-2 decimal places unless greater precision is analytically necessary.

Response: We agree. We simplified model-diagnostic reporting in the main text to two decimal places. For PFS, the consistency and UME DIC values are now reported as 17.50 and 17.49, and the uninformative four-decimal difference was removed. For OS, the values are 14.90 and 14.81 with a difference of 0.08. For confirmed ORR, the values are 34.33 and 34.26. Full numerical precision remains available in machine-readable analysis outputs where appropriate.

Location of revision: Results - Progression-free survival; Results - Overall survival; Results - Confirmed objective response rate.

5. Clarification of identical Lap-Cap versus PTN-Cap point estimates

Editor comment: Explain why the Lap-Cap versus PTN-Cap PFS HR is 2.66 in both the overall and second-line-only analyses even though the CrIs differ.

Response: We added the requested explanation immediately after the subgroup estimate. All trials contributing to the PTN-Cap comparison were second-line-only; therefore, the same evidence determines the central Lap-Cap versus PTN-Cap point estimate in the overall and second-line-only analyses. The CrIs differ because the subgroup-specific estimate is obtained within the restricted second-line-only network.

Location of revision: Results - Progression-free survival. Added sentence: “The identical Lap-Cap versus PTN-Cap point estimate in the overall and second-line-only analyses arose because all trials contributing to the PTN-Cap comparison were second-line-only; the different CrIs reflect estimation within the subgroup-specific network.”

6. Reference formatting

Editor comment: Correct Reference 22 to use “PLOS ONE” and check the entire reference list for consistent journal-name capitalization and PLOS style.

Response: We corrected Reference 22 from “PloS one” to “PLOS ONE” and performed a complete journal-name consistency check. We also standardized three visibly inconsistent journal-name entries to the same abbreviated style used throughout the reference list: “Lancet Oncol.” (Reference 5), “Cancer Drug Resist.” (Reference 24), and “J Transl Med.” (Reference 28). The bibliographic content, authors, years, volume/issue information, and DOIs were otherwise unchanged.

Location of revision: References 5, 22, 24, and 28.

7. Author affiliation check

Editor comment: Verify that Yu Xiao’s affiliation with the Sichuan Academy of Social Sciences is correct as typeset; if incorrect, update it, and if correct, confirm it in the response letter.

Response: We confirm that Yu Xiao’s affiliation with the Sichuan Academy of Social Sciences, Chengdu, Sichuan 610071, China, is correct as presented in the manuscript. No affiliation change is required.

Location of revision: Title page. The affiliation is retained as presented in the manuscript.

8. Final figure/table and Supporting Information cross-check

Editor comment: Perform a final proofread to ensure that all in-text callouts for Figures 1-5, Figures S1-S9, and appendices match the Supporting Information file list and legends, and correct minor inconsistencies before typesetting.

Response: We completed a global cross-check of the manuscript, standalone Figure Legends file, separate figure files, S1 materials, S2 Appendix, and revised S3 Appendix. Figures 1-5 and S1-S9 are all present with unique, continuous numbering and corresponding legends. The S3-S9 figure files were cross-checked against the corresponding files generated in the revised S3 package and were identical. The S2 Appendix table numbering and Supporting Information callouts were also checked. Minor legend wording/capitalization differences between the manuscript and standalone Figure Legends file were harmonized, including capitalization of “Convergence diagnostics” in the S3 Fig legend.

Location of revision: Figure Legends file; supporting-information legends in the manuscript; Figures 1-5 and S1-S9; S1-S3 Supporting Information materials.

Reviewer #2

We sincerely thank Reviewer #2 for the careful re-evaluation of our manuscript and for confirming that all comments from the previous round have been adequately addressed. We particularly appreciate the reviewer’s positive assessment of the technical soundness of the study, the rigor and appropriateness of the statistical analysis, the availability of the underlying data, and the clarity of the manuscript. No additional reviewer-specific revision was requested in this round.

We again thank the Academic Editor and Reviewer #2 for their careful and constructive evaluation. All requested editorial revisions have now been incorporated into the revised manuscript and Supporting Information. We respectfully submit the revised materials for further consideration.

Sincerely,

Gang Hu, on behalf of all authors

Attachment Submitted filename: Response to Editor.docx

# Editor report: Decision Letter 2

**DOI:** 10.1371/journal.pone.0358540.r005

**Authors:** Alireza Shoari

3 Sep 2026

Efficacy and Safety of T-DXd Versus Other HER2-Targeted Therapies in Second-Line and Later Settings for HER2-Positive Metastatic Breast Cancer: A Bayesian Network Meta-Analysis

PONE-D-26-23670R2

Dear Dr. Hu,

We’re pleased to inform you that your manuscript has been judged scientifically suitable for publication and will be formally accepted for publication once it meets all outstanding technical requirements.

Within one week, you’ll receive an e-mail detailing the required amendments. When these have been addressed, you’ll receive a formal acceptance letter and your manuscript will be scheduled for publication.

An invoice will be generated when your article is formally accepted. Please note, if your institution has a publishing partnership with PLOS and your article meets the relevant criteria, all or part of your publication costs will be covered. Please make sure your user information is up-to-date by logging into Editorial Manager at Editorial Manager® and clicking the ‘Update My Information' link at the top of the page. For questions related to billing, please contact billing support.

If your institution or institutions have a press office, please notify them about your upcoming paper to help maximize its impact. If they’ll be preparing press materials, please inform our press team as soon as possible -- no later than 48 hours after receiving the formal acceptance. Your manuscript will remain under strict press embargo until 2 pm Eastern Time on the date of publication. For more information, please contact onepress@plos.org.

Kind regards,

Alireza Shoari

Academic Editor

PLOS One

Additional Editor Comments (optional):

Reviewers' comments:

# Editor report: Acceptance letter

**DOI:** 10.1371/journal.pone.0358540.r006

**Authors:** Alireza Shoari

PONE-D-26-23670R2

PLOS One

Dear Dr. Hu,

I'm pleased to inform you that your manuscript has been deemed suitable for publication in PLOS One. Congratulations! Your manuscript is now being handed over to our production team.

At this stage, our production department will prepare your paper for publication. This includes ensuring the following:

* All references, tables, and figures are properly cited

* All relevant supporting information is included in the manuscript submission,

* There are no issues that prevent the paper from being properly typeset

You will receive further instructions from the production team, including instructions on how to review your proof when it is ready. Please keep in mind that we are working through a large volume of accepted articles, so please give us a few days to review your paper and let you know the next and final steps.

Lastly, if your institution or institutions have a press office, please let them know about your upcoming paper now to help maximize its impact. If they'll be preparing press materials, please inform our press team within the next 48 hours. Your manuscript will remain under strict press embargo until 2 pm Eastern Time on the date of publication. For more information, please contact onepress@plos.org.

You will receive an invoice from PLOS for your publication fee after your manuscript has reached the completed accept phase. If you receive an email requesting payment before acceptance or for any other service, this may be a phishing scheme. Learn how to identify phishing emails and protect your accounts at https://explore.plos.org/phishing.

If we can help with anything else, please email us at customercare@plos.org.

Thank you for submitting your work to PLOS One and supporting open access.

Kind regards,

PLOS One Editorial Office Staff

on behalf of

Dr. Alireza Shoari

Academic Editor

PLOS One
